# Integrating green chemistry with biomedical innovation: the role of biocompatible ionic liquids and ionic liquid nanoparticles in therapeutic applications

**DOI:** 10.1039/d5ra08137h

**Published:** 2026-02-02

**Authors:** Shoba Gunasekaran, Fahd A. Nasr, Mohammed F. Alotibi, Raghavi Rajasekar, Rakshitha Srinivasan, Mohammed Al-zharani, Fahad Ibrahim Alghuraybi, Tamizhdurai Perumal

**Affiliations:** a Department of Biotechnology, Dwaraka Doss Goverdhan Doss Vaishnav College 833, Gokul Bagh, E.V.R. Periyar Road, Arumbakkam Chennai 600106 Tamil Nadu India; b Biology Department, College of Science, Imam Mohammad Ibn Saud Islamic University (IMSIU) Riyadh 11623 Saudi Arabia; c Institute of Refining and Petrochemicals Technologies, King Abdulaziz City for Science and Technology (KACST) P.O. Box 6086 Riyadh 11442 Kingdom of Saudi Arabia mfalotaibi@kacst.gov.sa; d Institute of Advanced Materials, King Abdulaziz City for Science and Technology (KACST) P. O. Box 6086 Riyadh Saudi Arabia; e Department of Chemistry, Dwaraka Doss Goverdhan Doss Vaishnav College (Autonomous)(Affiliated to the University of Madras, Chennai) 833, Gokul Bagh, E.V.R. Periyar Road, Arumbakkam Chennai 600106 Tamil Nadu India

## Abstract

Biocompatible ionic liquids (ILs) have emerged as a new class of versatile materials that sit at the intersection of sustainability and biomedical innovation. Unlike traditional volatile organic solvents, ILs are salts that remain liquid at or near room temperature, offering distinctive features such as negligible vapor pressure, high thermal and chemical stability, broad solvation ability, and remarkable structural tunability. These properties have made them attractive as green solvents and catalysts, reducing environmental impact while improving reaction efficiency. More recently, attention has expanded from their conventional chemical roles to their potential as biologically active agents and functional materials. In particular, ionic liquid-based nanoparticles (ILNs) have opened new opportunities in medicine and biotechnology by combining the adaptable chemistry of ILs with the nanoscale advantages of controlled size, high surface area, and enhanced stability. ILNs have been shown to improve drug solubility and targeted delivery, enhance enzyme stabilization, and exhibit antimicrobial and anticancer activities. Moreover, task-specific and bio-ionic liquids (TSILs and B-ILs) can be precisely engineered to balance biocompatibility with functionality, positioning them as promising candidates for applications in tissue engineering, biosensing, and sustainable therapeutics. Despite these advances, issues related to cytotoxicity, biodegradability, and scalability remain challenges to be addressed. This review explores the properties of biocompatible ILs, their role in nanoparticle synthesis, and their expanding applications across green chemistry and therapeutic sciences, highlighting their potential to serve as a bridge between environmental responsibility and human health.

## Abstract 2

Biocompatible ionic liquids (ILs) are emerging materials linking sustainability and biomedicine. These room-temperature salts show negligible vapor pressure, high thermal and chemical stability, broad solvation power, and highly tunable structures.

## Graphical abstract

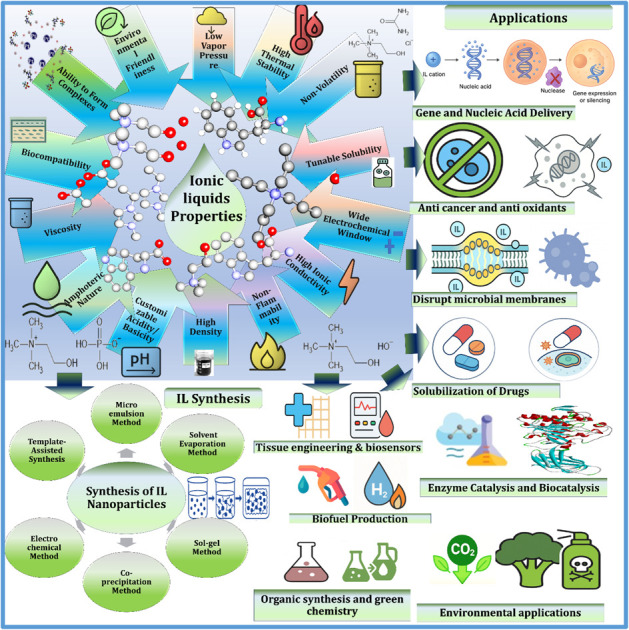

## 1. Introduction

The increasing demand for clean, sustainable methods in chemical research and industry highlights the growing environmental consciousness driving innovation. One of the most essential guidelines for sustainable chemical practices is the elimination of environmentally harmful organic solvents and the minimization of waste production during chemical synthesis.^1^ Traditional solvent-based processes often lead to toxic byproducts and substantial environmental impact, prompting the search for eco-friendly alternatives. In this context, ionic liquids (ILs) have emerged as a favorable solution due to their remarkable chemical and physical properties, which make them optimal candidates for green chemistry applications.^2,3^ Ionic liquids, which maintain a liquid state at low or ambient temperatures, are notable for their diverse and intriguing properties. They feature a wide liquid range, low volatility, excellent thermal stability, and can readily dissolve a variety of inorganic, organic, and polymeric substances.^4^ Unlike conventional solvents, ionic liquids have negligible vapor pressure, eliminating concerns about evaporation and the associated environmental impact. These properties make ILs particularly attractive as solvents in chemical reactions, offering significant advantages over traditional organic solvents.^5^

In particular, ionic liquids, especially those containing imidazolium cations, have been found to serve as effective media for catalytic organic processes. They not only enhance the reaction rate but also improve selectivity and facilitate catalyst recovery.^6^ The capacity of ILs to act both as solvents and catalysts in various reactions is a key factor in their growing popularity within green chemistry. The incorporation of ionic liquids in organic synthesis offers several benefits, including faster reaction rates, easier product recovery, and enhanced catalyst immobilization and recycling. Their ability to control product distribution further positions them as powerful tools for sustainable chemistry.^7,8^

A significant area of research has focused on the development of task-specific ILs, tailored by incorporating defined functional groups to address the unique requirements of particular reactions. These functionalized IL combine the benefits of homogeneous catalysis with the environmental advantages of ionic liquids. TSILs have demonstrated to be highly effective as catalysts in a range of chemical reactions, contributing to the innovation in sustainable and efficient chemical synthesis. The “designer” characteristics of ionic liquids provide for the fine-tuning of their characteristics, enhancing their versatile for a broad spectrum of applications. For example, ionic liquids can be engineered to possess acidic, basic, or neutral properties depending on the specific needs of the chemical reaction, offering enhanced control over reaction mechanisms.^9^

In recent years, research has expanded beyond their classical role as alternative solvents towards exploring ILs as bioactive agents, nanomaterials, and task-specific functional liquids. For example, ILs have demonstrated promising biological activity, including antimicrobial and anticancer effects, as well as the ability to stabilize enzymes, proteins, and nutraceutical compounds.^10,11^ This dual capacity serving as both functional solvents and therapeutic agents positions ILs at the interface of green chemistry and life sciences, offering solutions that address sustainability while advancing health-related technologies.^12^ Moreover, the development of ionic liquid-based nanoparticles (ILNs) and bio-ionic liquids (B-ILs) has opened new avenues for drug delivery, biosensing, tissue engineering, and agricultural applications.^6^

Given their versatility, ILs are increasingly recognized as a cornerstone in the design of sustainable, biocompatible, and multifunctional systems. However, challenges remain regarding cytotoxicity, biodegradability, and large-scale production, which must be tackled to achieve their maximum capability. This review endeavors to summarize the current understanding of ILs, focusing on their emerging roles as nanoparticles, biological agents, and green solvents, with an emphasis on enzyme stabilization, antimicrobial mechanisms, anticancer activity, biomedical applications, and environmental sustainability.

## 2. Classification of ionic liquids based on green chemistry and biological relevance

**Fig. 1:**
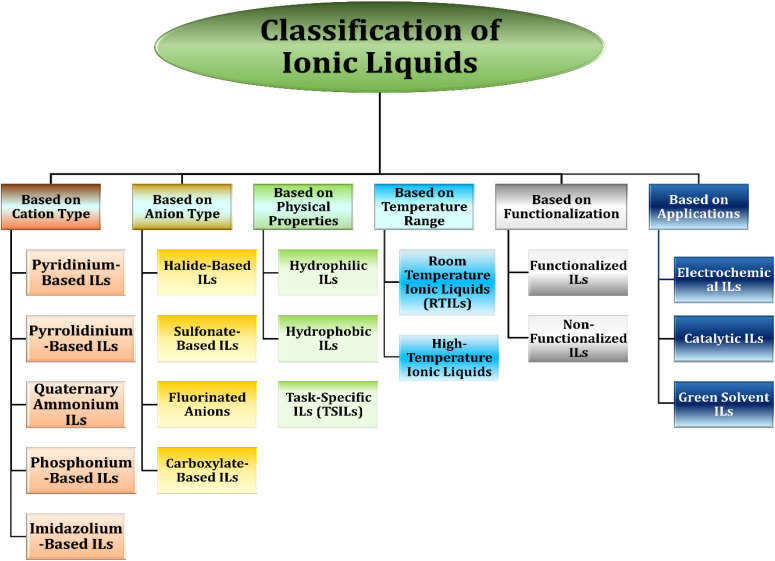
Classification of ionic liquids based on cation type, anion type, physical properties, temperature range, functionalization, and applications.^14^ Adapted from ref. 14 with permission from Elsevier, © 2023. License no. 6191350805273.

The classification of ionic liquids (ILs) can be broadly organized based on their structural components and functional properties (Fig. 1). Depending on the cation type, ILs include imidazolium, pyridinium, pyrrolidinium, quaternary ammonium, and phosphonium derivatives, each imparting distinct physicochemical characteristics. The anion type also strongly influences IL behavior, ranging from halide and sulfonate-based systems to fluorinated and carboxylate anions. ILs can further be distinguished by physical properties, such as hydrophilic, hydrophobic, or TSILs, designed for targeted applications. In terms of temperature range, ILs are divided into room-temperature ILs (RTILs) and high-temperature ionic liquids. Functionalization provides another layer of classification, separating functionalized ILs with reactive groups from their non-functionalized counterparts. Finally, based on applications, ILs are widely utilized as electrochemical media, catalytic supports, and green solvents due to their tunable properties and environmental benefits.^13^ This systematic classification underscores the versatility of ILs and their expanding role in sustainable technologies.

Ionic liquids (ILs) are a versatile group of compounds with unique physicochemical characteristics, making them use across diverse applications. Among the various types, imidazolium-based ILs are the extensively investigated owing to their excellent thermal stability, excellent ionic conductivity, and tunable properties, making them ideal for catalysis, electrochemical applications, and organic synthesis. Pyridinium-based ILs, derived from pyridine, exhibit remarkable thermal stability and electrochemical behavior, often employed in fuel cells, batteries, and as solvents for chemical reactions.^15^ Phosphonium-based ILs are notable for their exceptional thermal stability and hydrophobicity, finding applications in extraction processes, polymer synthesis, and lubrication.^16^ Choline-based ILs, being biocompatible and environmentally friendly, are particularly attractive for pharmaceutical and biomedical uses, including enzymatic reactions and drug delivery systems.^17^ Ammonium-based ILs, derived from ammonium cations, are valued for their low viscosity and high aqueous solubility, making them useful in separations, as electrolytes, and in green chemical processes. Piperidinium-based ILs demonstrate high thermal and chemical stability, supporting their use in electrochemical devices, supercapacitors, and as solvents in organic synthesis.^18^ In addition, TSILs are specifically engineered with targeted functional groups for specific applications such as catalysis, CO_2_ capture, and metal extraction, providing targeted and efficient performance in specialized processes. A related subclass, deep eutectic solvents (DES), resulting from hydrogen bond donor–acceptor association, are emerging as cost-effective and environmentally benign alternatives.^19^ DESs are increasingly applied in biocatalysis, biomolecule extraction, and as green solvents across various chemical industries. The diversity, adaptability, and sustainability of these ILs and DESs highlight their expanding role in modern scientific and industrial applications (Table 1). Ionic liquids with aromatic anions often exhibit increased toxicity due to their hydrophobicity and planar structures, which can disrupt cell membranes, interact with DNA or proteins, and induce oxidative stress. These ILs show higher toxicity toward microbes, aquatic organisms, and mammalian cells and tend to persist in the environment, highlighting the need for careful design to balance functionality with safety.^27^

**Table 1:** Overview of different types of ionic liquids, their characteristics, features, and applications

Type	Characteristics	Features	Applications	Ref.
Imidazolium-based ILs	High thermal stability, tunable hydrophobicity and hydrophilicity, strong ionic conductivity	Wide chemical versatility, high polarity	Antimicrobial agents, electrochemical applications, enzyme stabilization, organic synthesis	20
Pyridinium-based ILs	Aromatic structure, high chemical stability, enhanced antimicrobial properties	Can form π–π interactions, strong cation–anion interaction	Antimicrobial, catalytic processes, CO_2_ capture, biomass processing	21
Phosphonium-based ILs	High hydrophobicity, low viscosity, thermal and chemical stability	Biocompatibility with functionalization, task-specific tuning	Biofuels, CO_2_ capture, antimicrobial agents, enzymatic reactions	22
Choline-based ILs	Non-toxic, biodegradable, derived from renewable sources	Biocompatible, low environmental impact	Pharmaceuticals, biocatalysis, nutraceuticals, agriculture	23
Ammonium-based ILs	Wide range of hydrophobic and hydrophilic properties, moderate thermal stability	Flexible functionalization, antimicrobial properties	Antimicrobial agents, electrochemistry, lubricants, separation processes	24
Piperidinium-based ILs	Cyclic amine structure, high thermal stability, good solvent properties for polar compounds	Reduced toxicity, customizable side chains	Electrolytes for batteries, solvent systems, antimicrobial agents	25, and 26
Task-specific ILs (TSILs)	Custom-designed for specific reactions or applications (*e.g.*, catalysis, CO_2_ capture)	High efficiency in target applications, functionalized cation or anion	Catalysis, CO_2_ sequestration, selective extraction, pharmaceuticals	27
Deep eutectic solvents (DES)	Mixtures of hydrogen bond donors and acceptors with low melting points and biodegradable	Environmentally friendly, easy synthesis, tunable properties	Green chemistry, biocatalysis, pharmaceuticals, metal extraction, nanomaterial synthesis	28

**Fig. 2:**
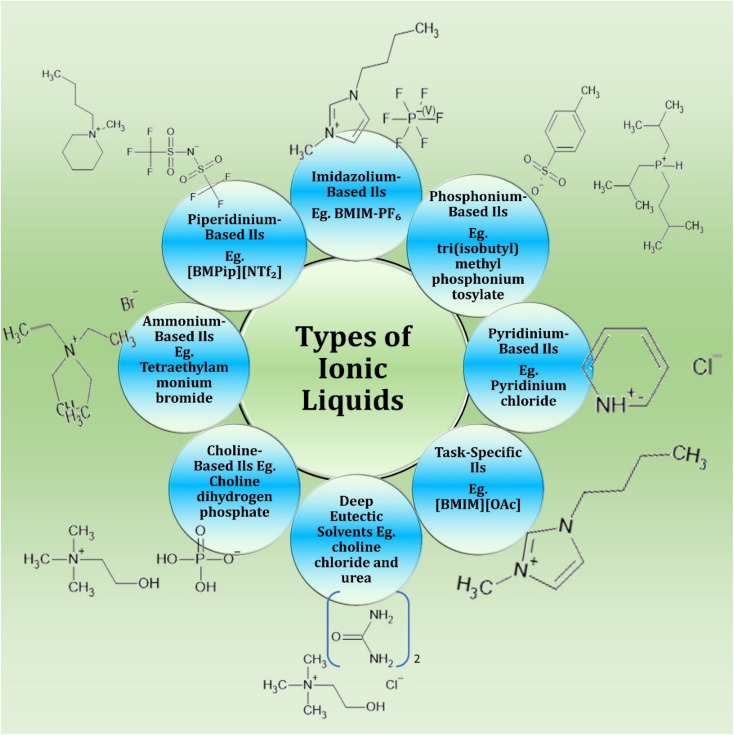
Representative types of ionic liquids (ILs) with selected examples.

Ionic liquids (ILs) can be systematically classified based on their cations, anions, and structural modifications, with representative examples illustrated in Fig. 2. Imidazolium-based ILs, namely 1-butyl-3-methylimidazolium hexafluorophosphate (BMIM-PF_6_, PubChem CID: 2734174), are widely investigated for their stability and solvent versatility. Pyridinium-based ILs, exemplified by pyridinium chloride (PubChem CID: 69401), demonstrate strong solvation capacity and potential antimicrobial applications. Phosphonium-based ILs, such as tri(isobutyl)methylphosphonium tosylate (PubChem CID: 10481282), are distinguished by exceptional thermal stability and catalytic utility. Task-specific ionic liquids (TSILs), like 1-butyl-3-methylimidazolium acetate ([BMIM][OAc], PubChem CID: 2734162), are engineered with functional groups for enhanced selectivity in catalysis and green chemical processes. Deep eutectic solvents (DES), such as the choline chloride (PubChem CID: 6209) and urea (PubChem CID: 1176) mixture, offer biodegradable, cost-effective alternatives to conventional solvents. Biocompatible choline-based ILs, for example choline dihydrogen phosphate (PubChem CID: 150067), are highly relevant in pharmaceutical and biotechnological applications. Ammonium-based ILs, including tetraethylammonium bromide (PubChem CID: 6285), provide versatility in solubility and conductivity, supporting biomedical and electrochemical uses. Finally, piperidinium-based ILs, such as *N*-butyl-*N*-methylpiperidinium bis(trifluoromethylsulfonyl)imide ([BMPip][NTf_2_], PubChem CID: 2368), are emerging as reduced-toxicity solvents with promising roles in energy storage and electrochemical devices.

Ionic liquids (ILs) represent a structurally diverse and functionally versatile group of compounds. Their classification is often based on the type of cation, which determines their physicochemical behavior, biological activity, and suitability for sustainable chemical applications. This section discusses the major classes of ILs and related DES, emphasizing their roles in green chemistry and biological systems.

### 2.1 Imidazolium-based ionic liquids

Imidazolium ILs are among the extensively studied, valued for their thermal stability, low viscosity, and broad solvating power.^29,30^ Their cationic framework supports hydrogen bonding and ionic interactions, making them excellent solvents for catalysis, organic synthesis, and electrochemical applications.^31^ However, the acidic C_2_-hydrogen can deprotonate under basic conditions, generating carbenes that complicate catalytic processes.^32^ Biologically, imidazolium ILs demonstrate antimicrobial and biofilm-disrupting activities, particularly when developed as polymeric ILs such as poly(1-vinyl-3-ethylimidazolium) bromide, which combine reduced cytotoxicity with strong antimicrobial efficacy.^33,34^ In green chemistry, they serve as sustainable substitutes for volatile organic solvents and stabilize enzymes in biocatalytic processes, highlighting their dual industrial and biomedical significance.

### 2.2 Pyridinium-based ionic liquids

Pyridinium ILs (PILs) are characterized by π–π interactions, ionic conductivity, and thermal stability.^35^ In green chemistry, they are utilized as recyclable solvents and catalysts in selective synthesis and extraction of bioactive compounds, heavy metals, and dyes. Their conductivity also makes them effective electrolytes for batteries, supercapacitors, and fuel cells.

Biologically, PILs exhibit antimicrobial and antiviral effects, disrupting bacterial membranes and viral envelopes.^33,36^ Certain PILs show selective cytotoxicity against tumor cells, offering potential for anticancer therapy.^37^ However, cytotoxicity and production costs remain barriers, reinforcing the need for designing biodegradable, low-toxicity PILs.

### 2.3 Phosphonium-based ionic liquids

Phosphonium ILs are distinct for their exceptional thermal stability (>400 °C), making them ideal for high-temperature reactions such as hydroformylation, Heck, and Suzuki coupling.^38^ They also function as phase-transfer catalysts and are increasingly applied in CO_2_ capture, polymerization, and radical-based chemistry.^39^

Their biological activities include antimicrobial, antifungal, and anticancer effects, often through membrane disruption and reactive oxygen species (ROS) generation. Their dual role as green solvents and bioactive agents positions them as promising candidates in both sustainable industry and medical innovation.

### 2.4 Choline-based ionic liquids

Choline-based ILs, obtained from a naturally occurring nutrient, are biodegradable, biocompatible, and non-toxic, aligning strongly with green chemistry principles. They offer excellent solvation ability for both polar and non-polar compounds and are applied in drug delivery, enzyme stabilization, nutraceutical extraction, and sustainable agriculture.^40^

Their amphiphilic structure enhances biomolecule stability and bioavailability while minimizing toxicity, making them especially suited for pharmaceutical and biotechnological applications.

### 2.5 Ammonium-based ionic liquids

Ammonium ILs are among the oldest studied, with ethanolammonium nitrate first reported in 1888.^41^ They exhibit tunable hydrophobicity/hydrophilicity, strong solvation power, and ionic conductivity, enabling their use in bioseparations, electrochemical devices, and antimicrobial coatings.^42^ In green chemistry, they support low-energy processes, separation technologies, and electrolyte systems, while biologically they show antimicrobial activity and compatibility in pharmaceutical formulations. Their adaptability highlights their role in sustainable industrial and medical technologies.

### 2.6 Piperidinium-based ionic liquids

Piperidinium ILs, defined by their cyclic amine cation, offer high thermal stability, strong polarity, and reduced toxicity compared to many other ILs.^43^ Their tunable side chains enable customization for electrochemical applications, particularly as electrolytes in lithium-ion batteries, where structural design influences solvation and ionic conductivity.

Biologically, they are being explored for antimicrobial and therapeutic applications, while their eco-friendly profile and efficiency in high-temperature processes make them attractive for green solvent technologies.

### 2.7 Task-specific ionic liquids

TSILs are engineered ILs with functional groups tailored for specific functions, such as catalysis, selective extraction, or antimicrobial action.^44^ In green chemistry, they excel in metal extraction (*e.g.*, Co^2+^, Ni^2+^), CO_2_ capture, and catalytic selectivity.^45^

Biomedically, functionalized imidazolium or phosphonium TSILs can stabilize enzymes, inhibit microbial enzymes, and improve drug delivery, offering targeted efficiency with reduced side effects.^46^ Their dual catalytic and therapeutic roles highlight their transformative potential in both sustainable chemistry and medicine.

### 2.8 Deep eutectic solvents (DES)

DES, while distinct from ILs, share many IL-like features including reduced volatility, thermal robustness, and tunable solubility. They generated by combining quaternary ammonium salts (*e.g.*, choline chloride) with hydrogen bond donors (*e.g.*, urea, alcohols, acids).^47^

In green chemistry, DES are used in metal processing, electroplating, nanomaterial synthesis, and biomass conversion.^28^ Their low toxicity and biodegradability make them especially appealing for biomolecule extraction, drug formulations, and pharmaceutical applications. Challenges such as high viscosity remain, but their low cost and eco-friendliness make DES an emerging alternative to traditional ILs.

### 2.9 Ionic liquids as nanoparticles

Traditionally regarded as liquid salts at ambient conditions, ionic liquids (ILs) have emerged as versatile templates and stabilizers in nanoparticle synthesis, attributed to their unique physicochemical characteristics. Their low volatility and high heat stability, and tunable solubility make ILs highly effective in controlling nanoparticle size, shape, and stability. ILs can act as both solvents and morphology-directing agents, facilitating the formation of nanoparticles with well-defined morphologies. In catalysis, IL-derived nanoparticles exhibit enhanced increased efficiency as a result of their large surface area, stability, and functional versatility. ILs also enable the distribution of nanoparticles, minimizing agglomeration and maintaining active surface sites. Beyond catalysis, ILs have been utilized in biomedical applications, where they serve as vehicles for nanoparticle therapeutic delivery platforms. Their biologically compatible nature and ability to stabilize nanoparticles in aqueous and non-aqueous environments make them suitable for targeted delivery and controlled release of therapeutics. Moreover, IL-stabilized nanoparticles have shown potential in energy storage, strong CO_2_ affinity, sensing, and environmental remediation.^48^ The ability to tune the ionic environment by selecting specific cation–anion pairs allows for the customization of nanoparticle properties to suit diverse applications. As research progresses, the integration of ILs in nanoparticle synthesis and application is expected to open new avenues in nanotechnology and material science.

**Fig. 3:**
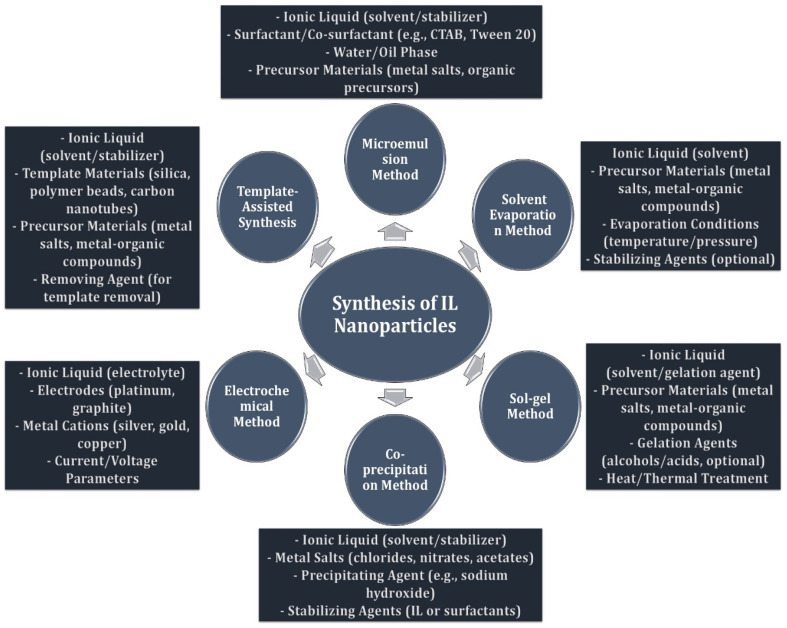
Schematic representation of ionic liquids as nanoparticles and common methods of synthesis.

Ionic liquid nanoparticles (ILNPs) are a rapidly developing class of materials known for their unique properties, including high surface area, tunability, and the ability to interact with both organic and inorganic materials. Several methods exist for synthesizing ILNPs, such as solvent-based synthesis, co-precipitation, electrostatic assembly, microemulsion, sol–gel, and laser ablation techniques (Fig. 3). In solvent-based synthesis, ionic liquids act as stabilizing agents or solvents, helping to prevent nanoparticle agglomeration and control size. The co-precipitation method involves the formation of nanoparticles by reacting metal salts with bases in the presence of ionic liquids, while electrostatic assembly leverages ionic liquid interactions to stabilize or coat nanoparticles. Microemulsion techniques use ionic liquids as surfactants to form microdroplets that act as nanoreactors for nanoparticle growth. In sol–gel processes, ionic liquids are used as solvents or templates for metal oxide nanoparticles, and laser ablation in ionic liquids provides a method for producing nanoparticles by irradiating a target material with high-energy lasers.^49^ These ILNPs are gaining attention for their utilization in variety of fields, namely catalysis, drug delivery, environmental remediation, energy storage, and sensors. In catalysis, ILNPs act as catalysts or catalyst supports, enhancing reaction efficiency and selectivity, while in drug delivery, they improve the solubility and bioavailability of hydrophobic drugs.^50^ For environmental remediation, ILNPs are used to remove pollutants from water or soil. In energy storage devices like supercapacitors and batteries, ILNPs enhance performance and longevity, and in sensor applications, they are employed to detect specific molecules or environmental changes. Due to their stability, tunable properties, and versatility, ILNPs are poised to exhibit a key role in advancing technologies across various industries.

#### 2.9.1 Solvent evaporation method

The solvent evaporation method is a extensively employed for synthesizing nanoparticles, including ionic liquid nanoparticles (ILNPs), by exploiting the evaporation of a solvent to induce nanoparticle formation.^51^ This method involves dissolving the material to be synthesized (such as a metal precursor or polymer) in a solvent (often an ionic liquid or organic solvent) to form a solution. The solution is then exposed to controlled environmental conditions, typically by placing it in a vacuum or allowing it to evaporate under ambient conditions, which causes the solvent to gradually evaporate, facilitating the generation of nanoparticles.

In the context of ionic liquid nanoparticles, the IL acts as both the solvent and stabilizing agent, preventing the aggregation of nanoparticles as they form. The evaporation process can be modulated to influence the size, shape, and distribution of the nanoparticles. This approach provides numerous benefits, including simplicity, large-scale applicability, and the capacity to produce nanoparticles with narrow size distributions. Ionic liquids used in this process can enhance solubility, minimize particle agglomeration, and ensure structural stability to the nanoparticles, which is crucial for achieving consistent and high-quality products.^52^

The solvent evaporation method is particularly advantageous for producing nanoparticles in a diverse applications, including drug delivery where the nanoparticles can be engineered for controlled release, or in catalysis, where the nanoparticles can be designed for specific catalytic activities. Additionally, by selecting appropriate ionic liquids and solvent evaporation conditions, it is possible to tailor the surface characteristics of the nanoparticles, which can impact their interactions with biological systems or other materials.^53^

While the solvent evaporation method is relatively straightforward, it still faces challenges, such as the need for precise control over solvent evaporation rates and the potential for solvent residues that may affect the final nanoparticle product. However, ongoing advancements in the utilization of ionic liquids in this process are expected to enhance the efficiency and versatility of the solvent evaporation method, making it a valuable tool for the production of advanced nanoparticles with a broad range of applications.

#### 2.9.2 Sol–gel method

The sol–gel method technique for synthesizing IL-based nanoparticles, particularly metal oxide nanoparticles.^54^ This methodology begins with the synthesis of a sol, where metal salts or metal–organic compounds are dissolved in an ionic liquid to form a homogeneous liquid phase. Ionic liquids serve as an ideal solvent in this method due to their high stability, tunable solubility, and ability to stabilize nanoparticle formation. The sol undergoes gelation, where gelation agents such as alcohols, acids, or bases are added to induce the organization into a 3D interconnected nanoparticle structure. The gel is then aged to further consolidate its structure before undergoing drying to remove the solvent. In some cases, thermal treatment or calcination is employed to remove any residual organic components and to crystallize the nanoparticles.^55^ The final product consists of well-formed nanoparticles with controlled size and morphology, which can be tailored by adjusting the concentration of ionic liquid, precursor materials, and processing conditions. The sol–gel method, in combination with ionic liquids, offers several advantages, including precise control over particle size, high purity, eco-friendliness, and stability.^56^ This method is used in catalysis, sensors, energy storage, and environmental remediation for metal oxide nanoparticles' unique properties.

#### 2.9.3 Co-precipitation method

Ionic liquid (IL)-assisted co-precipitation has emerged as an effective method for synthesizing superparamagnetic Fe_3_O_4_ nanoparticles with controlled size, morphology, and high dispersion.^57,58^ In this approach, nanoparticles are precipitated from an ionic liquid solution by adding a co-solvent, which reduces their solubility and promotes formation. The method typically involves reacting ferric chloride (FeCl_3_) and ferrous sulfate (FeSO_4_) with a base such as sodium hydroxide (NaOH) in the presence of an IL, such as 1-butyl-3-methylimidazolium chloride ([Bmim]Cl), which acts as a stabilizing agent.^59^

The IL plays a crucial role in regulating nucleation and growth, providing electrostatic and steric stabilization that prevents aggregation and ensures a narrow size distribution. TEM analyses reveal that the resulting Fe_3_O_4_ nanoparticles are spherical with uniform diameters of 10–15 nm, while XRD confirms a well-defined magnetite crystalline structure. VSM measurements demonstrate superparamagnetic behavior at room temperature, a desirable property for biomedical applications, including targeted drug delivery and magnetic resonance imaging (MRI), as well as environmental remediation. Superparamagnetic nanoparticles do not retain magnetization in the absence of an external field, allowing controlled guidance without aggregation.^60^

Compared to traditional methods, IL-assisted co-precipitation offers several advantages. The approach allows precise control over particle size and morphology, ensures uniform dispersion, and leverages the tunable physicochemical properties of ILs, such as polarity, viscosity, and solubility, to optimize synthesis. Additionally, ILs are environmentally friendly, recyclable, and can facilitate scale-up for industrial applications.

Despite these advantages, further research is needed to assess the long-term stability, biocompatibility, and environmental impact of ILs in nanoparticle synthesis. Optimization of synthesis parameters—including temperature, IL concentration, and reaction time—could further enhance nanoparticle properties. Overall, IL-assisted co-precipitation provides a versatile and scalable strategy for producing well-dispersed, superparamagnetic Fe_3_O_4_ nanoparticles with tailored properties suitable for applications in catalysis, drug delivery, MRI, hyperthermia therapy, and environmental remediation.^61^

#### 2.9.4 Electrochemical deposition

Electrochemical techniques have emerged as an effective approach for depositing ionic liquids (ILs) as nanoparticles onto surfaces, enabling the fabrication of well-defined nanostructures.^62^ ILs, which are salts in the liquid state at ambient temperatures, offer a sustainable alternative to traditional organic solvents commonly used in electrolytes. Their low volatility, non-flammability, and tunable physicochemical properties make them particularly attractive for electrochemical processes.

The use of ILs in electrodeposition provides enhanced control over the morphology, size, and crystallinity of deposited nanomaterials. Imidazolium- and pyrrolidinium-based ILs have been shown to stabilize metal ions in solution, prevent nanoparticle aggregation, and facilitate uniform growth of nanostructures. This enables the synthesis of highly ordered nanomaterials with improved electrochemical performance. Metals such as gold, copper, and silver have been successfully electrodeposited using ILs, demonstrating the versatility of this approach for applications in catalysis, sensors, and energy storage devices.^63^

IL-based electrodeposition offers several advantages over conventional methods, including precise control over nanoparticle size and shape, high deposition rates, and the ability to produce materials with unique properties. Moreover, the elimination of volatile organic solvents reduces environmental impact and waste generation, highlighting the sustainability of this approach.

Despite these benefits, further research is needed to optimize IL composition and electrodeposition parameters, as well as to evaluate the scalability of these processes for industrial applications. Overall, ILs provide a promising platform for the green and controlled synthesis of advanced nanomaterials with tailored properties for diverse technological applications.^64^

#### 2.9.5 Microemulsion or nanoreactor method

Ionic liquids can be used in microemulsions as nanoreactors to synthesize nanoparticles. The ionic liquid phase stabilizes the nanoparticles and controls their size and morphology.^65^ These are versatile systems with applications in areas like drug delivery, catalysis, and material synthesis. Microemulsions are thermodynamically stable mixtures of water, oil, and surfactant, often with a co-surfactant, that form nanoscale droplets and exhibit unique properties, such as enhanced solubilization and improved transport characteristics. The incorporation of ionic liquids into microemulsions has gained significant attention due to their ability to modify the physicochemical properties of these systems, enhancing their stability and functionality. The paper provides a comprehensive overview of the various types of ionic liquids that have been successfully integrated into microemulsions, including both imidazolium-based and phosphonium-based ILs, and discusses how their unique characteristics, such as high polarity, tunable solubility, and ability to interact with both the aqueous and organic phases, contribute to the formation and stabilization of microemulsions.^66^ The authors highlight the importance of selecting appropriate surfactants and co-surfactants, as these play a crucial role in the formation of stable microemulsions with ILs. They also discuss the challenges associated with the design of microemulsion systems containing ILs, particularly in terms of achieving a balance between the IL's properties and the stability of the microemulsion. The paper explores the impact of ionic liquids on the droplet size, phase behavior, and overall stability of the microemulsion, noting that ILs can significantly alter the interfacial tension between the oil and water phases, which in turn affects the size and distribution of the droplets. The authors further investigate the characterization techniques used to study microemulsions with ionic liquids, such as dynamic light scattering (DLS), small-angle X-ray scattering (SAXS), and transmission electron microscopy (TEM), which provide insights into the size, morphology, and structural organization of the microemulsion droplets. The paper also discusses the potential applications of IL-containing microemulsions, particularly in the fields of nanomaterial synthesis, where these systems can act as templates for the formation of nanoparticles, and in catalysis, where the ILs can enhance the catalytic activity and stability of the microemulsion-based systems. Additionally, the authors highlight the environmental and sustainability aspects of using ionic liquids in microemulsions, emphasizing that ILs can offer greener alternatives to traditional solvents and surfactants. Despite the promising results, the paper acknowledges that there are still challenges to be addressed, including the potential toxicity and environmental impact of certain ionic liquids, as well as the need for further optimization of the formulation and characterization techniques to fully exploit the potential of IL-based microemulsions. In conclusion, the paper provides a thorough examination of the role of ionic liquids in the formulation and characterization of microemulsions, highlighting their ability to enhance the stability and functionality of these systems and offering insights into their diverse applications in nanotechnology, catalysis, and other fields.^67^

#### 2.9.6 Template-assisted synthesis

Template-assisted synthesis is a method used to create nanoparticles with well-defined sizes, shapes, and structures by utilizing a template to guide the formation of the nanoparticles. In this process, the template serves as a mold or scaffold to direct the assembly of the nanoparticle material. The template could be a porous material, such as silica, polymer beads, or carbon nanotubes, which is coated with the material of interest (*e.g.*, metal salts, metal–organic compounds, or ionic liquids). Once the material is deposited or synthesized around the template, the template is removed, leaving behind the desired nanoparticle structure.^68^

This technique is particularly useful for creating nanoparticles with intricate or controlled shapes, such as hollow spheres, rods, or tubes, which are difficult to achieve using other synthesis methods. The use of templates allows for precise control over the size and morphology of the nanoparticles, making it an attractive approach for applications requiring highly specific nanoparticle characteristics.^69^

The process typically involves several key steps: (1) template preparation, where the template material is synthesized or selected based on the desired nanoparticle shape; (2) material deposition, where the precursor material is introduced to the template and allowed to form around it; (3), often achieved through chemical etching, calcination, or dissolution, leaving the desired nanoparticle structure; and (4) nanoparticle characterization, where the resulting particles are analyzed for their size, shape, and other properties.^70^

Ionic liquids (ILs) can be incorporated into template-assisted synthesis as both a solvent and a stabilizing agent, providing a controlled environment for nanoparticle formation. ILs help in preventing aggregation, stabilizing the nanoparticles, and enhancing the overall performance of the synthesized material. The template-assisted synthesis method is highly versatile and can be applied in various fields such as catalysis, drug delivery, sensors, and energy storage.

### 2.10 Properties of ionic liquids and ionic liquid-based nanoparticles (ILNs)

Ionic liquids (ILs) are a class of molten salts that remain liquid below 100 °C and have gained significant attention due to their extraordinary physicochemical properties. One of the most attractive features of ILs is their low vapor pressure and non-volatility, which minimizes environmental emissions and makes them safer alternatives to volatile organic solvents. Their high thermal stability allows use under harsh processing conditions, while non-flammability enhances safety in industrial and laboratory applications.^71^

The tunable solubility of ILs, arising from the vast diversity of possible cation–anion combinations, enables selective dissolution of organic, inorganic, and biomolecular substrates. In electrochemical applications, ILs are valued for their wide electrochemical window and high ionic conductivity, making them suitable electrolytes for batteries, supercapacitors, and fuel cells.

Beyond these features, ILs also exhibit high density and viscosity, which can be advantageous or limiting depending on the application. Their amphoteric nature and customizable acidity/basicity allow for fine-tuning of reactivity, particularly in catalysis and chemical synthesis. Certain ILs also demonstrate biocompatibility, opening avenues in drug formulation, enzyme stabilization, and biotransformation processes. Furthermore, their ability to form molecular complexes enhances applications in separation science, extraction, and nanomaterials.^72^

**Fig. 4:**
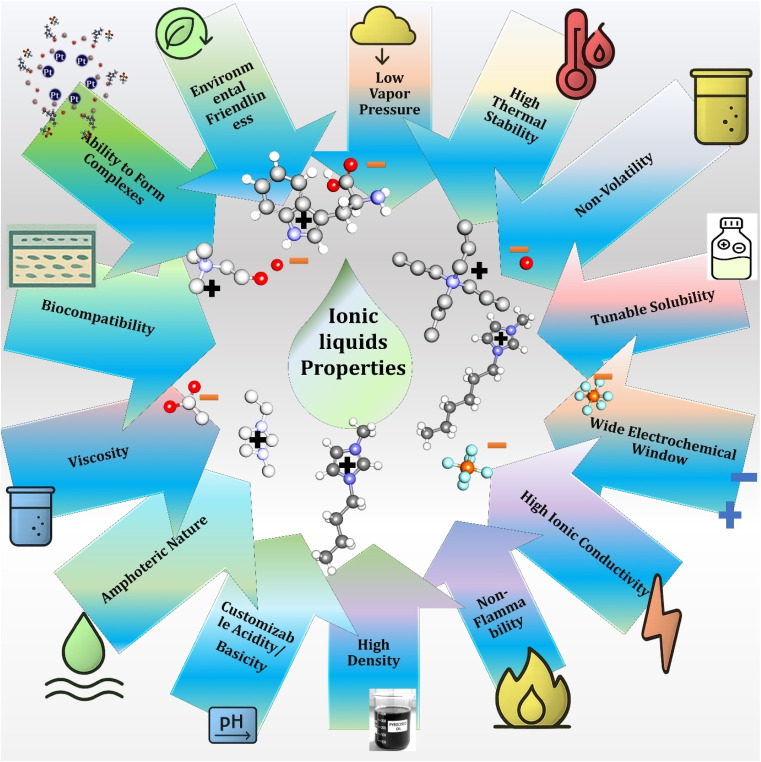
Key physicochemical and functional properties of ionic liquids (ILs).

Importantly, the design of ILs with environmental friendliness in mind—using biodegradable and less toxic cations and anions—positions them as promising candidates for sustainable technologies. Overall, the multifunctional properties of ILs make them powerful tools across green chemistry, materials science, electrochemistry, biotechnology, and energy applications, highlighting their transformative potential in both fundamental research and industrial practice. Ionic liquids possess a wide range of physicochemical features such as low vapor pressure, high ionic conductivity, tunable solubility, and environmental friendliness (Fig. 4).

Ionic liquid-based nanoparticles (ILNs) exhibit a variety of tunable properties that make them highly versatile for applications across catalysis, drug delivery, sensing, energy storage, and environmental remediation. These properties can be broadly categorized into structural, surface, optical/thermal/magnetic, and stability characteristics.^73^ The major tunable characteristics of ILNs, including their structural, surface, and physicochemical properties, are summarized in Table 2.

**Table 2:** Key properties of ionic liquid-based nanoparticles (ILNs)

Property	Description	Key features/Implications	References
Structural properties (size, shape, morphology)	Controlled size (nm–µm), tunable shape (spherical, rod-like, complex), defined morphology	Smaller size → higher surface area; shape affects reactivity, drug loading, catalytic activity; morphology affects performance in energy storage, environmental, biomedical applications	74
Surface characteristics (charge, hydrophilicity/hydrophobicity)	Surface charge determined by IL cation/anion	Charged surfaces → stability & dispersion; functionalization → targeted delivery, biocompatibility, enhanced catalysis	75
	Hydrophilic or hydrophobic surfaces adjustable *via* IL choice or functionalization		
Optical, thermal, magnetic properties	Optical: fluorescence, absorption, scattering; thermal: high thermal stability	Tunable optical behaviors for imaging/biosensing; withstands high temperatures → catalysis & energy storage; magnetic ILNs → targeted drug delivery, MRI, magnetic separation	76
	Magnetic: magnetically responsive when metal/metal oxide incorporated		
Stability	High chemical, thermal, and colloidal stability due to IL characteristics (low volatility, high solubility, low vapor pressure)	Maintains performance under extreme conditions (temperature, pressure, corrosive environments); critical for industrial and catalytic applications	76

#### 2.10.1 Structural properties (size, shape, and morphology)

Ionic liquid-based nanoparticles (ILNs) typically have controlled sizes ranging from a few nanometers to microns, depending on the synthesis method and the desired application. The ability to control the size is essential for optimizing their performance in applications such as catalysis, drug delivery, and sensing. Smaller-sized nanoparticles often have a higher surface area, which enhances their reactivity and functionality. The shape of ILNs can be easily modified through various synthesis techniques. They can adopt spherical, rod-like, or more complex geometries, allowing for tuning of their properties. The shape of the nanoparticles directly affects their surface area, interaction with other molecules, and their behavior in different environments. For example, anisotropic shapes may exhibit enhanced catalytic activities or improved drug loading and release profiles. The morphology of ILNs refers to the overall structure, including their surface roughness and internal organization. The morphology is influenced by factors such as the ionic liquid's properties, the solvent used, and the synthesis conditions. A well-controlled morphology helps optimize the performance of ILNs in a wide range of applications, including energy storage, environmental remediation, and biomedical uses.^74^

#### 2.10.2 Surface characteristics (surface charge, hydrophilicity/hydrophobicity)

The surface charge of ILNs is determined by the cation and anion of the ionic liquid. The surface can be either positively or negatively charged, which influences the stability of the nanoparticles in solution and their interaction with other materials. A charged surface also affects the dispersion behavior and can be used for targeted delivery or magnetic separation in biomedical and environmental applications.^77^ The hydrophilic or hydrophobic nature of the surface can be adjusted by modifying the ionic liquid or adding functional groups to the surface. Hydrophilic ILNs interact well with water, making them suitable for aqueous-based applications such as drug delivery and biological sensing. Hydrophobic ILNs, on the other hand, are more appropriate for use in non-aqueous solvents or for applications requiring water-insensitive surfaces. ILNs can be functionalized with various chemical groups, such as amino, thiol, or carboxyl groups, to enhance their interaction with specific substrates or biological systems. This surface modification allows for precise control over the nanoparticles' behavior, such as improving biocompatibility for drug delivery or enhancing their reactivity in catalysis. The ability to easily modify the surface is one of the key advantages of ILNs in diverse applications, from sensors to biomedical therapies.^78^

#### 2.10.3 Optical, thermal, and magnetic properties

ILNs can exhibit unique optical characteristics, such as fluorescence, absorption, and scattering, depending on their size and shape. These properties can be fine-tuned for applications in imaging, biosensing, or optoelectronics. Quantum size effects, such as photoluminescence, can be observed in smaller nanoparticles, leading to tunable optical behaviors. The thermal stability of ILNs is enhanced due to the inherent stability of ionic liquids. They are resistant to thermal degradation, allowing them to withstand high temperatures. This property is particularly useful in applications such as catalysis, energy storage, and thermal management, where nanoparticles need to maintain stability under elevated temperatures.^79^ Some ILNs are designed to be magnetically responsive, especially when magnetic metal or metal oxide nanoparticles are incorporated into the ionic liquid matrix. These magnetic properties allow for applications in targeted drug delivery, magnetic resonance imaging (MRI), and magnetic separations. The magnetization of ILNs can be controlled by adjusting the size, composition, and shape of the nanoparticles.^80^

#### 2.10.4 Stability of ionic liquid-based nanoparticles

ILNs are highly stable due to the unique characteristics of ionic liquids, such as low volatility, high solubility in both organic and aqueous solvents, and low vapor pressure. These properties make ILNs ideal for use in harsh environments, such as extreme temperatures, high pressure, or corrosive conditions. Their stability is also critical in industrial applications, where they are exposed to rigorous processing conditions. The ability to maintain stability in diverse conditions makes ILNs a versatile material for many fields, including catalysis, material science, and energy storage.^76^ In summary, ionic liquid-based nanoparticles possess a range of tunable properties, including size, shape, surface charge, and stability, which make them highly versatile for a wide range of applications. Their unique combination of structural flexibility, surface modification capabilities, and stability in extreme conditions positions them as valuable materials in fields like catalysis, drug delivery, and environmental remediation.^75,77^ ILs are highly stable (both thermally and chemically), making them effective excipients in drug formulations; they can improve the solubility of poorly water soluble drugs by enabling specific interactions (hydrogen bonds, ionic interactions, π–π stacking) with the drug molecules.^78^ In drug active ILs (API ILs), where the drug itself is paired with suitable ions, researchers have demonstrated dramatically enhanced solubility (*e.g.*, hundreds of times higher for drugs like diclofenac) and improved stability, mediated *via* hydrogen bonding and van der Waals forces.^79^

## 3. Applications of ionic liquids in biological systems and green chemistry

Ionic liquids are chemical substances with applications in various areas of technology and science. The extraordinary properties of these materials may be modified by adjusting the cation and anion ratio, making it realistic and specific to their needs. ILs are becoming more prevalent among ecologists, medical researchers and biochemists due to their strong biological activity, in addition to their chemical and physical features.^81^ These compounds have been developed for biological uses such as antibacterial and cytotoxic properties, as well as drug transport and production.^58^ In recent years, the use of ILs as an alternative reaction media has gained interest and widespread use in drug discovery technologies, including traditional organic solvents.^82^

**Fig. 5:**
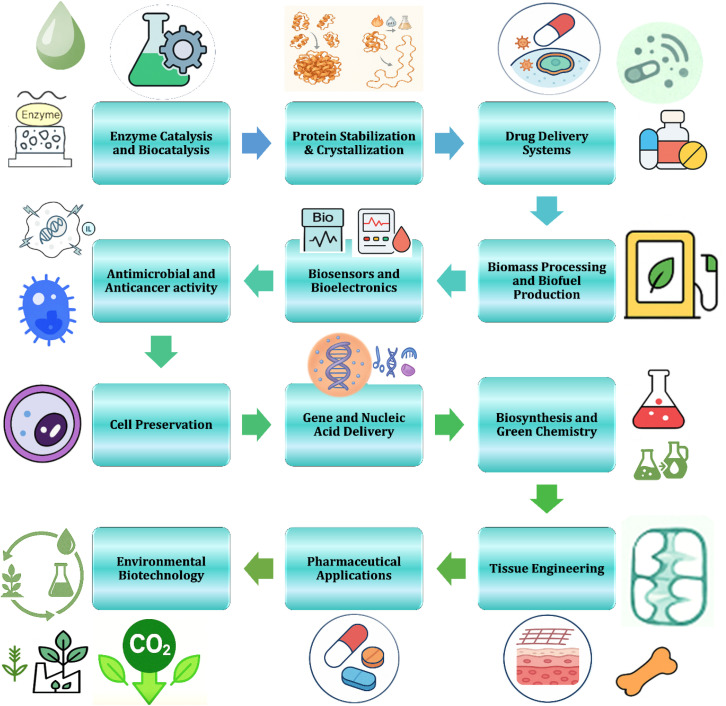
Schematic representation of the diverse applications of ionic liquids. Adapted from ref. 83 under the terms of the Creative Commons license.

Ionic liquids (ILs) have found diverse applications in both biomedicine and green chemistry, as illustrated in Fig. 5. In biocatalysis, ILs enhance enzyme stability and activity, enabling sustainable synthetic processes.^84^ Their role in protein stabilization and crystallization has been particularly valuable for structural biology and pharmaceutical formulation.^85^ ILs are also widely applied in drug delivery systems, where their tunable polarity improves solubility and bioavailability of poorly soluble drugs.^86^ Antimicrobial and anticancer activities of ILs, especially imidazolium and phosphonium derivatives, are attributed to their membrane-disruptive and cytotoxic effects.^46,87^ Moreover, ILs are integrated into biosensors and bioelectronics due to their ionic conductivity and biocompatibility.

In the energy and sustainability domain, ILs support biomass processing and biofuel production by acting as solvents for lignocellulosic biomass,^88^ and they enable green catalysis through task-specific design for selective transformations. Biologically, ILs aid in cell preservation and gene/nucleic acid delivery due to their ability to form stable complexes with biomolecules. Furthermore, ILs are being explored for biosynthesis, tissue engineering using silk fibroin dissolution, and pharmaceutical innovations, including controlled drug release.^89^ Finally, their biodegradability and low volatility support environmental biotechnology applications such as CO_2_ capture and sustainable agriculture.^90^

### 3.1 Enzyme stabilization and catalysis in ionic liquids

**Fig. 6:**
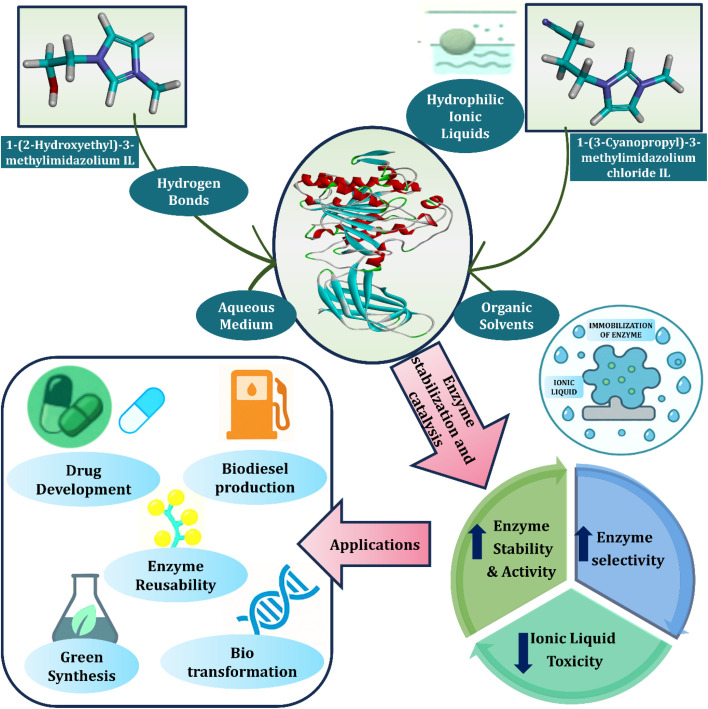
Role of ionic liquids in enzyme stabilization and catalysis. Partially reproduced and modified from ref. 91.

Ionic liquids (ILs), particularly functionalized imidazolium and pyridinium ILs, have become significant tools in enhancing enzyme activity and stability, making them ideal candidates for various biotechnological applications. Hydrophilic ionic liquids, such as 1-(2-hydroxyethyl)-3-methylimidazolium IL and 1-(3-cyanopropyl)-3-methylimidazolium chloride IL, interact with enzymes through hydrogen bonds in aqueous and organic solvents. These interactions enhance enzyme stability, activity, and selectivity while reducing ionic liquid toxicity. Immobilization of enzymes in ionic liquids enables broad applications, including drug development, biodiesel production, green synthesis, enzyme reusability, and biotransformation (Fig. 6).

Functionalized imidazolium ILs, such as [C_2_OHmim]^+^ (1-(2-hydroxyethyl)-3-methylimidazolium) and [C_3_CNmim]^+^ (1-(cyanopropyl)-3-methylimidazolium), are known for their biocompatibility and ability to stabilize enzymes. The hydroxyl group in [C_2_OHmim]^+^ forms hydrogen bonds with enzymes, preventing denaturation and stabilizing their structure. The hydrophilic nature of [C_3_CNmim]^+^ minimizes deep membrane interactions, reducing toxicity while maintaining antibacterial activity, making these ILs particularly suitable for enzyme catalysis.^92^ These ILs are also used in various biotechnological processes, such as drug development, biodiesel production, and bio-based chemical synthesis, where enzyme stability is crucial.

Choline-based ILs, such as choline chloride-urea, form deep eutectic solvents (DES), which have been shown to enhance lipase activity for biodiesel synthesis, offering an eco-friendly alternative to conventional organic solvents.^93^ The use of DES systems aligns with green chemistry principles by reducing environmental impact while enhancing enzyme activity in non-aqueous media.

Task-specific ionic liquids (TSILs) also play an important role in catalysis by providing unique environments for chemical transformations, stabilizing reactive intermediates, and improving reaction rates and selectivity.^94^ These ILs can be tailored to support specific catalytic processes, including those in green chemistry, where sustainability and waste reduction are key goals.^95^

Acidic ionic liquids, formulated with anions like chloride (Cl−), bromide (Br−), and bis(trifluoromethylsulfonyl)imide (NTf2^−^), are particularly useful in catalytic processes. They influence properties such as solubility, viscosity, and thermal stability, which are essential for efficient catalysis in sustainable chemical systems.^94^

The application of ionic liquids supported on solid carriers, such as silica or alumina, facilitates continuous catalytic processes in industrial settings. These systems enhance scalability, sustainability, and efficiency by improving reaction consistency and reducing downtime in large-scale applications.^96^

Polymeric supports like poly(4-vinylpyridine) and poly(1-vinylimidazole) stabilize ionic liquids, prevent their leaching, and ensure efficient catalytic cycles. These supports help maintain the catalytic activity of ILs while improving their reusability in industrial catalysis.^97^

In biocatalysis, lyophilized enzymes (free enzymes) often face challenges in ILs due to low solubility and activity. Hydrophilic ILs can remove enzyme-bound water molecules, which are necessary for enzyme function, potentially reducing catalytic activity.^98^ However, advances in solvent engineering and enzyme formulation are being developed to optimize enzyme systems in ILs for biotransformations.^99^

Ionic liquids have also been shown to improve biotransformations involving hydrophilic or polar substrates that are insoluble or slightly soluble in organic solvents. ILs serve as effective agents for enzyme immobilization, enhancing enantioselectivity, stability, and catalytic activity in non-aqueous environments.^100^ IL-coated enzymes have shown superior catalytic performance and reusability compared to free enzymes.^101^

Recent advancements in enzyme encapsulation with ILs have demonstrated their potential in producing highly active and stable biocatalysts for non-aqueous biotransformations. IL-coated enzymes exhibit superior catalytic activity, stability, and enantioselectivity, making them promising candidates for biotransformations in challenging conditions.^101^

The use of ionic liquids, particularly functionalized imidazolium, pyridinium, and choline-based ILs, has opened new opportunities for enzyme stabilization and catalysis in biotechnological applications. These ILs provide a unique environment for enhancing enzyme activity and stability, making them suitable for a wide range of applications in green chemistry, biocatalysis, and industrial processes. However, challenges related to enzyme solubility and activity in ILs require ongoing research to optimize their use in large-scale biotransformations.

### 3.2 Antimicrobial mechanisms and applications of ionic liquids (ILs)

Ionic liquids (ILs) are gaining prominence as antimicrobial agents due to their unique physicochemical properties, which enable them to disrupt microbial cell membranes, induce oxidative stress, and interact with cellular components. These properties make ILs valuable in diverse medical, industrial, and environmental applications. The antimicrobial mechanisms of ILs vary depending on their chemical structure, and different types of ILs, including imidazolium, pyridinium, phosphonium, ammonium, and piperidinium-based ILs, offer a broad spectrum of biological activities. Homo-DILs based on 1-alkyl-3-methylimidazolium units have shown broad antimicrobial activity, effectively inhibiting multiple airborne bacterial, yeast, and fungal pathogens.^102^ IL-modified gold nanoparticles with tailored anions enable precise control over lysozyme binding, antimicrobial activity, and thermal stability, establishing IL-based surface engineering as a powerful strategy for advanced enzyme immobilization.^103^

**Fig. 7:**
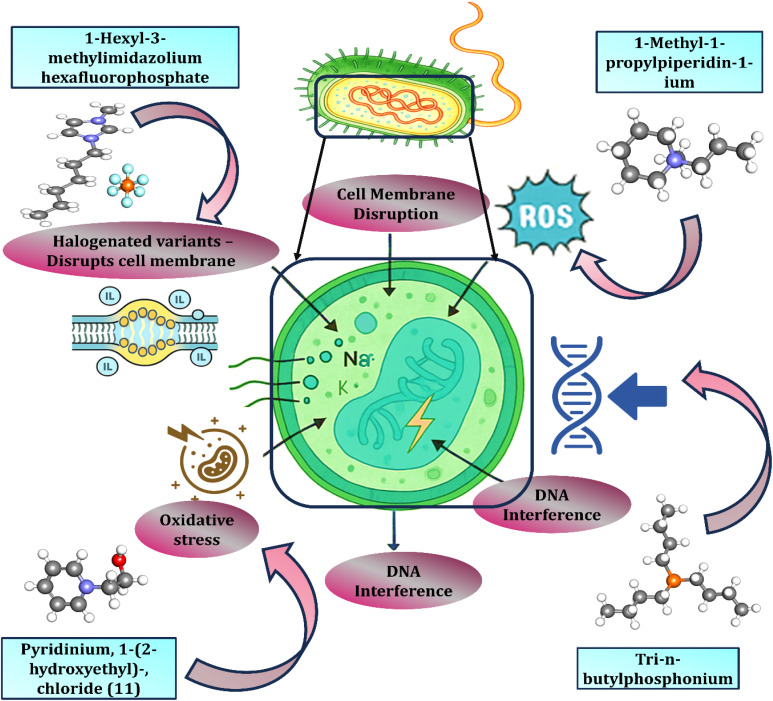
Antimicrobial mechanisms of ionic liquids (ILs) showing cell membrane disruption, oxidative stress induction, reactive oxygen species (ROS) generation, and DNA interference, leading to microbial cell death. Partially reproduced and modified from ref. 104, licensed under Creative Commons Attribution (CC BY).

Schematic representation of the antimicrobial action of ionic liquids (ILs): different classes of ILs, including imidazolium-, piperidinium-, pyridinium-, and phosphonium-based compounds, exert antimicrobial effects through multiple mechanisms, such as disruption of microbial cell membranes, inhibition of biofilm formation, and interference with cellular metabolic processes. These include disruption of microbial cell membranes, induction of oxidative stress and reactive oxygen species (ROS) generation, as well as interference with intracellular targets such as DNA.^104^ The combined effects of these mechanisms compromise microbial viability, ultimately leading to cell death (Fig. 7).

#### 3.2.1 Imidazolium-based ILs

Imidazolium-based ILs, particularly those with shorter alkyl chains like 1-butyl-3-methylimidazolium [C_4_mim]^+^, are known for their strong antimicrobial properties.^105^ These ILs typically disrupt microbial membranes by interacting with lipid bilayers, leading to ion leakage and cell lysis. The hydrophobic nature of the alkyl chain plays a critical role in the antimicrobial activity, with shorter chains being particularly effective in membrane disruption. Longer alkyl chains, such as those in 1-hexyl-3-methylimidazolium ([C_6_mim]^+^) and 1-dodecyl-3-methylimidazolium ([C_12_mim]^+^), enhance hydrophobic interactions, leading to stronger antimicrobial and cytotoxic effects.^106^ Halogenated imidazolium ILs, like 1-(4-chlorobutyl)-3-methylimidazolium ([C_4_Clmim]^+^) and 1-ethyl-3-(2-bromoethyl)imidazolium ([C_2_Brmim]^+^), exhibit increased reactivity, which enhances their ability to damage microbial DNA and cell walls.^107^ IL-mediated citrate reduction produced stable AgNPs with distinct sizes and structures, and among them, Ag-[BMIM][OTf] showed the strongest antibacterial activity, achieving low inhibitory concentrations against *E. coli*, *S. aureus*, and *E. cloacae*, highlighting the role of ILs in tuning nanoparticle antimicrobial performance.^108^ Midazolium-based ionic liquids showed strong antibacterial and antibiofilm effects against multidrug-resistant *E. coli* causing urinary tract infections, while maintaining low cytotoxicity and good biocompatibility, demonstrating their promise as alternative therapeutic agents.^109^ These ILs have promising applications as disinfectants, preservatives, and antimicrobial coatings, offering a more sustainable alternative to conventional antimicrobial agents.^110^

#### 3.2.2 Pyridinium-based ILs

Pyridinium-based ILs, such as 1-butylpyridinium and 1-hexylpyridinium, demonstrate significant antimicrobial and antifungal activities, primarily through the disruption of cell membranes, leading to ion leakage and cell lysis.^111^ The length of the alkyl chain influences their antibacterial properties, with longer chains making them more effective against both Gram-positive and Gram-negative bacteria. These ILs have found applications in medical fields, including wound healing agents and topical disinfectants. Halogenated pyridinium ILs, such as 1-butyl-3-chloropyridinium, show enhanced antiviral properties due to the chlorine atom, which increases their reactivity, allowing them to disrupt microbial cell walls and interfere with DNA functions. These ILs have the potential to combat resistant bacterial strains and viruses. Additionally, 1-ethyl-3-bromopyridinium generates oxidative stress, increasing reactive oxygen species (ROS) production, which damages microbial DNA and impedes viral replication.^112^

#### 3.2.3 Phosphonium-based ILs

Phosphonium-based ILs are recognized for their broad-spectrum antimicrobial activities. Trialkylphosphonium ILs disrupt microbial membranes through hydrophobic interactions, leading to ion leakage and cell lysis. These ILs also induce oxidative stress, which contributes to their antimicrobial effects. Phosphonium ILs hold potential for cancer therapy, where they could selectively target cancer cells.^113^ Tetraalkylphosphonium ILs exhibit antifungal properties, disrupting fungal membranes and preventing cellular function, which makes them potential solutions for combating fungal infections.^114^ Functionalized phosphonium ILs can stabilize enzymes and disrupt microbial cell walls, offering promising applications in both industrial enzymatic processes and medical treatments.^115^

Halogenated phosphonium ILs, which contain chlorine or bromine, exhibit enhanced antimicrobial properties by disrupting cell walls and interfering with microbial DNA. These ILs can also disrupt biofilms and inhibit fungal growth, further enhancing their antimicrobial potential.^116^ Fluorinated phosphonium ILs generate ROS, destabilize microbial membranes, and induce apoptosis in cancer cells, making them suitable for both antimicrobial and anticancer applications.^117^ Polymeric phosphonium ILs prevent microbial adhesion and biofilm formation, making them particularly useful in medical devices and industrial applications where biofilm-related infections are common.^118^

#### 3.2.4 Ammonium-based ILs

Ammonium-based ILs, such as benzalkonium-based ILs, are effective antimicrobial agents that work by disrupting microbial membranes, leading to cell lysis. These ILs are widely used in medical disinfectants, coatings, preservatives, and industrial applications, including water treatment.^119^ Their low toxicity to human tissues makes them suitable for use in sensitive environments, offering an eco-friendly alternative to traditional chemical disinfectants.

#### 3.2.5 Selective and biocompatible pyridinium ILs

Functionalized pyridinium-based ILs, such as 1-(2-hydroxyethyl)pyridinium, have demonstrated selective antimicrobial activity, targeting microbial cell membranes while exhibiting low toxicity to human cells. This biocompatibility makes them particularly suitable for applications in pharmaceuticals, tissue engineering, and medical devices.^120^ Amino acid-based ionic liquids, particularly those formed with imidazolium or choline cations, are emerging as green and biocompatible solvent alternatives, though studies such as Guncheva *et al.* (2015) indicate they may reduce protein thermal stability.^121^ These properties make pyridinium ILs excellent candidates for various biomedical applications, offering selective antimicrobial effects while minimizing adverse effects on human cells.

#### 3.2.6 Piperidinium-based ILs

Piperidinium-based ILs also exhibit antimicrobial properties, making them valuable in sterilization and disinfection processes, particularly in healthcare and industrial settings.^122^ Their low toxicity makes them suitable for use in products that come into contact with human skin and sensitive environments. Anandkumar *et al.* (2020) the tested piperidinium-based IL (IL-M) that effectively inhibited planktonic cells of *Pseudomonas* spp., while the imidazolium-based fluoride-containing IL (IL-E) showed superior broad-spectrum performance by not only suppressing planktonic growth but also preventing bacterial adhesion and significantly reducing biofilm formation on titanium and carbon steel surfaces.^123^

#### 3.2.7 Biodegradable ionic liquids (ILs)

Biodegradable ILs are becoming increasingly important for sustainable applications. These ILs are designed to degrade more easily in the environment, addressing concerns related to the persistence and bioaccumulation of conventional ILs. Biodegradable imidazolium-based ILs, for example, have been shown to effectively target microbial membranes while minimizing toxicity to human cells.^124,125^ They are also being explored for use in drug delivery systems, where their controlled release properties can enhance the efficacy of treatments.^126^

Similarly, biodegradable phosphonium-based ILs are being developed for pharmaceutical applications.^127^ These ILs offer biocompatibility and antimicrobial properties, making them ideal candidates for addressing microbial threats with minimal side effects.^128^ Their potential in biomedical applications is significant, as they provide a sustainable approach to combating infections while aligning with green chemistry principles.^129^

The diverse antimicrobial properties of ILs—spanning across imidazolium, pyridinium, phosphonium, ammonium, and piperidinium families—underscore their potential as effective agents for combating bacterial, fungal, and viral infections.^130^ These ILs disrupt microbial membranes, generate ROS, and interfere with DNA functions, offering promising applications in medical, industrial, and environmental fields. Biodegradable ILs, in particular, provide a more sustainable approach to antimicrobial treatments, paving the way for innovative applications in healthcare and environmental management, and offering an eco-friendly alternative to traditional antimicrobial agents.

### 3.3 Ionic liquids in protein interaction and bioactive compound extraction

Proteins, one of the most versatile classes of biomolecules, exhibit diverse behaviors due to their complex structures and interactions. Their multiple phases and aggregation states arise from an extensive potential energy landscape, as well as their ability to bind other biomolecules such as lipids and carbohydrates. Active sites and metal prosthetic groups further contribute to their functional complexity.

Ionic liquids (ILs), particularly room-temperature ionic liquids (RTILs), have emerged as important additives to modulate protein characteristics in aqueous solutions or as solvents that can replace water and volatile organic compounds. The interaction of RTILs with proteins is complex, influenced by the structural diversity of both ILs and proteins.^131^ Factors such as amino acid sequence, secondary structure (α-helix, β-sheet), and the hydrophilic or hydrophobic nature of RTILs can significantly affect protein behavior. For instance, IL cations and anions may interact with specific amino acid residues, while the net charge of proteins, dependent on pH, also plays a crucial role. Despite these insights, the diversity of RTILs makes it challenging to establish predictive models for RTIL-protein interactions, especially when considering higher-order protein structures.^132^

Beyond proteins, ILs—particularly choline- and ammonium-based variants—have proven invaluable in the extraction and stabilization of bioactive compounds from natural sources, with significant applications in the nutraceutical sector. Choline-based ILs, such as choline chloride-glycerol, effectively extract polyphenols, flavonoids, and other antioxidants, achieving high yields and purity while enhancing stability and bioavailability.^133^ Ammonium-based ILs, such as triethylammonium acetate, are increasingly employed in separation processes to selectively extract bioactive compounds and remove impurities in pharmaceutical and nutraceutical industries ammonium-based ionic liquids improved betanin extraction from red beet juice (70–82%), demonstrating a promising green alternative to synthetic dyes.^134^ These ILs offer superior selectivity, enhanced solubility, and reduced environmental impact compared to traditional organic solvents, supporting sustainable and eco-friendly extraction practices.

Overall, ILs play a dual role: modulating protein interactions and enabling efficient, sustainable extraction of bioactive compounds, thereby contributing to advances in biotechnology, nutraceutical development, and green chemistry.

### 3.4 Anti-cancer activity of ionic liquids (ILs): mechanisms and applications

Ionic liquids (ILs) have garnered considerable attention in recent years for their potential applications in various fields, including anticancer therapies. The unique properties of ILs, such as their ability to dissolve a wide range of compounds and their tunable structures, make them promising candidates for drug development. This review discusses the cytotoxicity, anticancer mechanisms, and applications of ILs, highlighting their advantages and challenges in oncology.

Various classes of ionic liquids have been studied for their anticancer activity, with distinct mechanisms ranging from ROS generation to membrane disruption. For instance, imidazolium-based ILs like [BMIM][Cl] show strong apoptotic effects in breast and colon cancer cells, while amino acid-functionalized ILs demonstrate enhanced selectivity for liver and colorectal carcinoma.^135^ Examples of ionic liquids, their mechanisms of anticancer activity, and cancer types shown in Table 3.

**Table 3:** Examples of ionic liquids, their mechanisms of anticancer activity, and cancer types

IL type	Example	Mechanism	Cancer type (s)	References
Imidazolium-based	[BMIM][Cl]	ROS generation, apoptosis	MCF-7, MDA-MB-231, HCT116	136
Pyridinium-based	[HPy][Br]	ROS, cell cycle disruption	A549, HL-60	137
Phosphonium-based	[P_66614_][Cl]	Mitochondrial collapse, apoptosis/necrosis	PC-3, HeLa	138
Guanidinium-based	Dodecylguanidinium chloride	Membrane disruption, caspase activation	B16–F10, U87	139
Amino acid-functionalized	[BMIM][Gly], [BMIM][Ala]	Redox modulation, selective apoptosis	HepG2, SW480	140

#### 3.4.1 Cytotoxicity of ionic liquids

The cytotoxicity of ILs has been widely studied using assays like the MTT assay, which measures cell viability by detecting the reduction of MTT tetrazolium dye by mitochondrial enzymes.^96^ The cytotoxic effects of ILs can range from micromolar to millimolar concentrations, depending on their chemical structure and the cell type involved. ILs containing imidazolium, pyridinium, and phosphonium cations have been found to exhibit varying levels of toxicity toward tumor and normal cells.^138^

One of the key factors affecting IL cytotoxicity is the structure of the IL, which influences its interaction with cellular components. For example, imidazolium-based ILs tend to demonstrate higher toxicity in tumor cells compared to normal cells, but this effect is often nonselective. Some studies have shown that adding natural amino acids to ILs can increase their cytotoxicity, potentially due to the enhanced uptake of the toxic anions by cells.^138^ This suggests that the toxic effects of ILs are influenced by both their cationic and anionic components, as well as the environmental and cellular context.

#### 3.4.2 Mechanisms of cytotoxicity

ILs exert their cytotoxic effects through several mechanisms, including the induction of oxidative stress, DNA damage, and apoptosis. Studies have demonstrated that ILs such as those containing imidazolium cations can cause the generation of reactive oxygen species (ROS), which lead to oxidative damage within cells. ROS have been implicated in the initiation of apoptosis, a process that selectively targets cancer cells for programmed cell death.^137^ Additionally, fluorescence microscopy and specific lethality assays have been used to monitor the cellular effects of ILs, revealing significant DNA damage and cell cycle disruption.^139^

In particular, ILs with long alkyl chains, such as guanidinium-based ILs, have been shown to exhibit strong cytotoxicity against various tumor cell types. These ILs can effectively disrupt cellular membranes, leading to the leakage of vital cellular components. The cytotoxicity of ILs is also influenced by their ability to interact with cellular membranes and internalize into cells, where they can exert toxic effects on cellular structures and organelles.

**Fig. 8:**
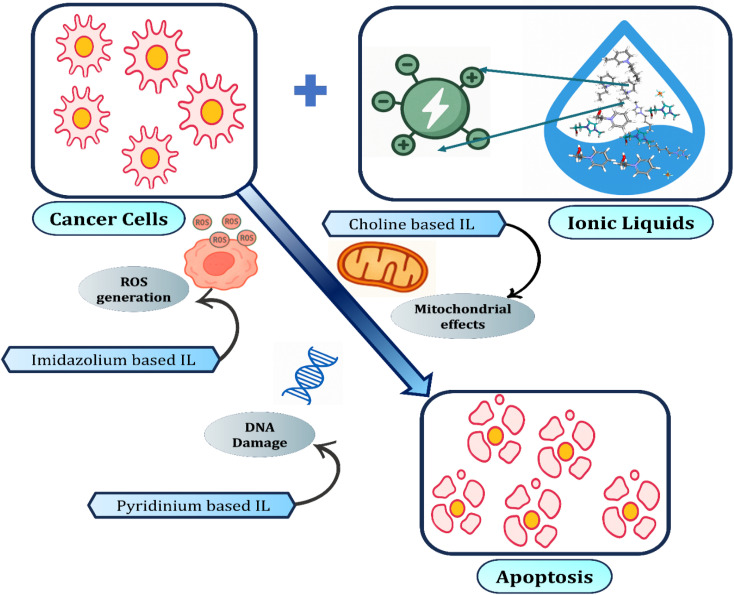
Anticancer mechanisms of ionic liquids (ILs). Partially reproduced and modified from ref. 142. Copyright © 2023 Elsevier B. V. Reproduced with permission under License no. 6191370203284.

Ionic liquids interact with cancer cells through multiple pathways leading to apoptosis. Imidazolium-based ILs promote reactive oxygen species (ROS) generation, while pyridinium-based ILs cause DNA damage. Choline-based ILs exert mitochondrial effects, disrupting energy metabolism. Collectively, these interactions trigger programmed cell death (apoptosis), highlighting the potential of ILs in cancer therapy. The anticancer activity of ionic liquids (ILs) is often attributed to their ability to induce oxidative stress, disrupt cellular membranes, and trigger apoptosis. The specificity toward cancer cells arises primarily from differences in membrane composition, metabolic rate, and redox state between cancerous and normal cells. Cancer cells generally have higher metabolic activity and elevated reactive oxygen species (ROS) levels, making them more susceptible to oxidative stress induced by ILs. Additionally, the structural tunability of ILs allows selective targeting by optimizing hydrophobicity, cation–anion combinations, or conjugation with cancer-targeting ligands (Fig. 8).^141^

#### 3.4.3 ILs in anticancer applications

Ionic liquids have shown great potential as anticancer agents. One of the key advantages of ILs in cancer therapy is their ability to selectively target cancer cells while minimizing damage to healthy cells. Pyridinium-based ILs, for example, have been shown to induce the generation of ROS, which selectively triggers apoptosis in cancer cells while sparing normal cells.^137^ This selective mechanism is particularly advantageous in cancer treatment, as it allows for more precise targeting of tumor cells, reducing the side effects commonly associated with traditional chemotherapy. Pandiyan *et al.* demonstrated that an imidazolium-based ionic liquid could function as both a stabilizing and templating agent in the green synthesis of ZnO-based hybrid nanostructures, with the resulting Ag–Au/ZnO nanoparticles (20–25 nm) showing enhanced antibacterial performance and significant anticancer activity, highlighting their potential applicability in biomedical nanotechnology.^143^

Moreover, ILs can be designed to have both anticancer and biocompatible properties. For example, imidazolium-based ILs have demonstrated significant cytotoxicity against human cancer cell lines while showing lower toxicity toward nonmalignant cells, such as human embryonic kidney (HEK) cells.^136^ This selectivity makes ILs promising candidates for the development of targeted anticancer therapies that could potentially reduce the adverse effects seen with conventional chemotherapies.

#### 3.4.4 Challenges and future directions

Despite the promising anticancer potential of ILs, several challenges remain in their development as therapeutic agents. One of the major hurdles is the lack of a comprehensive understanding of the mechanisms through which ILs exert their anticancer effects. Research has shown that the cytotoxicity of ILs can vary significantly depending on factors such as the specific type of cancer, the molecular structure of the IL, and the environmental conditions within the tumor microenvironment.^139^ As such, further studies are needed to identify the precise molecular targets of ILs and to develop predictive models for evaluating their anticancer efficacy.

Additionally, the biocompatibility of ILs with human cells and tissues must be thoroughly assessed before they can be considered for clinical applications. While some ILs have shown lower toxicity to normal cells, the long-term effects of exposure to ILs remain unclear. There is also a need for studies that investigate the pharmacokinetics and pharmacodynamics of ILs, including their absorption, distribution, metabolism, and excretion in the body.^144^

Ionic liquids hold great promise as anticancer agents due to their unique properties, including selective cytotoxicity, the ability to generate ROS, and their biocompatibility. While ILs have demonstrated significant potential in preclinical studies, further research is required to fully understand their mechanisms of action and to optimize their efficacy and safety for clinical use. By addressing the challenges associated with their cytotoxicity and biocompatibility, ILs could become a valuable tool in the development of targeted, less toxic anticancer therapies in the future.

### 3.5 Biomedical applications of ionic liquids (ILs): advancements in drug delivery and therapeutics

Ionic liquids (ILs), composed entirely of organic cations and anions, have become a focal point in biomedical research due to their distinct physicochemical properties, such as low melting points, ionic conductivity, non-volatile nature, and tunability. These properties position ILs as highly versatile compounds for advancing drug delivery systems, tissue engineering, electrochemical biosensors, and pharmaceutical formulations.^11^ This review explores the expanding role of ILs in biomedical applications, particularly their impact on drug delivery, tissue regeneration, biosensors, and medical device enhancement.

#### 3.5.1 Drug delivery systems and therapeutics

ILs have emerged as promising candidates in drug delivery systems (DDS) due to their ability to enhance solubility, stability, and bioavailability of drugs, particularly those that are poorly water-soluble. Their unique properties allow for controlled and targeted drug release, making them ideal for delivering anticancer agents and antibiotics.^145^ For instance, ILs can be incorporated into polymer-based hybrid materials to enhance drug stability and solubility, improving therapeutic efficacy and minimizing side effects.

A key advantage of ILs in drug delivery is their ability to selectively interact with microbial membranes, enhancing the therapeutic effects of antimicrobial drugs while reducing toxicity to human cells. For example, biodegradable phosphonium-based ILs have been shown to interact with bacterial membranes, making them suitable for infection control while minimizing cytotoxicity to human cells.^129^ Additionally, ILs can serve as solubilizing agents for poorly soluble drugs, enhancing their absorption and providing more efficient drug delivery. Ionic liquids (ILs) are increasingly explored in drug delivery due to their ability to enhance solubility of poorly water-soluble drugs, stabilize biomolecules, and be tailored for safety. Careful structural design has produced ILs that are both effective and biocompatible.

Cholinium-based ILs, derived from the nutrient choline, are particularly promising. Petkovic *et al.* (2010) showed they were well tolerated by microbial and mammalian cells,^146^ and Li *et al.* (2022) demonstrated enhanced protein stability without affecting cell viability.^147^ Jesus *et al.* (2019) reported that biocompatible *N*-acetyl amino acid *N*-alkyl cholinium ILs increased drug solubility up to fourfold while remaining non-toxic.^148^

However, Guncheva *et al.* (2019) highlighted a trade-off between stabilization and cytotoxicity: [Chol][Glu] enhanced insulin stability most, whereas [Chol][Asp] was least toxic.^149^ These findings emphasize the importance of balancing stabilization efficiency with biological safety, confirming that carefully designed ILs can provide both stability and low toxicity for pharmaceutical use.

The controlled release properties of ILs also enable the development of systems that can release drugs in response to specific stimuli, such as pH or temperature, allowing for more precise treatment regimens. Furthermore, task-specific ILs (TSILs), which are designed for specific applications, can provide tailored solutions for targeted drug delivery, minimizing unwanted side effects.

#### 3.5.2 Tissue engineering

ILs have shown significant promise in tissue engineering, particularly in the regeneration of natural fibers such as silk fibroin (SF). ILs such as [EMIM]Cl can dissolve silk fibroin at high concentrations without causing degradation, opening the possibility for creating silk-based materials for medical applications. These materials can be used for medical sutures, scaffolds, and composites designed for bone tissue engineering, drug release, and artificial skin.^85^

The ability of ILs to modify the mechanical, chemical, and physical properties of silk fibroin makes them valuable in tissue engineering applications. The enhanced water retention, mechanical properties, and stability of silk-based materials in adverse environments, such as *in vitro* cultures or *in vivo* conditions, make them ideal candidates for regenerative medicine. Moreover, ILs contribute to the promotion of cell growth and differentiation, which are crucial for tissue regeneration.^150^

#### 3.5.3 Electrochemical biosensor applications

In addition to their role in drug delivery and tissue engineering, ILs are playing a crucial role in the development of electrochemical biosensors. Their high ionic conductivity, electrochemical stability, and thermal stability make them excellent candidates for use in various electrochemical devices, including batteries, capacitors, and fuel cells. ILs can enhance the sensitivity and selectivity of sensors by acting as electrolytes, improving their performance in detecting specific analytes.^151^

ILs have been successfully used in ion-selective electrodes (ISEs), where their interactions with conducting polymers, such as poly(3,4-ethylenedioxythiophene) (PEDOT), have led to the creation of solid-state electrochemical sensors. These sensors demonstrate excellent response to both cations and anions, making them highly useful in applications such as gas–liquid chromatography and MALDI-MS. Furthermore, ILs can stabilize enzymes and other macromolecules, promoting the development of more robust and long-lasting biosensors.^152^

ILs are also used to create ionogels—materials with customizable ionic properties—that can be designed for specific applications, such as creating mesoporous or nanoporous structures for biosensor matrices. These ionogels show promise in improving the performance and stability of biosensors, leading to more sensitive and reliable diagnostic tools. The properties and applications of various ionic liquid (IL)-based platforms for biosensing are summarized in Table 4, highlighting the target molecules, electrode or biosensor types, and specific applications.

**Table 4:** Summary of ionic liquid (IL)-based platforms for biosensing applications: target molecules, electrode/biosensor types, and specific applications

Ionic liquid (IL)/platform	Target molecule	Electrode/biosensor type	Application	References
MWCNT/TOAI_3_/CILE (tetra-*n*-octyl ammonium triiodide)	Cysteine, ascorbic acid, dopamine	Carbon ionic liquid electrode	Detection in human serum & pharmacological samples	153
IL with enzyme cofactor + SWCNT bucky gel	Glucose	Multifunctional gel-based electrode	Diabetes monitoring	154
Chitosan/nano-V_2_O_5_/MWCNT composite + CILE	DNA (Yersinia enterocolitica LAMP product)	Carbon ionic liquid electrode	Pathogen detection	155
IL/mesoporous carbon/protein composite	Protein detection	Microelectrode	General biosensing	156
Ionic liquid-modified graphite	microRNA-34a	Impedimetric biosensor	Genetic biomarker detection	157
Biocompatible IL/graphene-chitosan/hemoglobin	Nitromethane	Electrochemical sensor	Environmental pollutant detection	158
Co nanoparticles/CILE	Myoglobin	Electrochemical biosensor	Clinical biomarker detection	159
Myoglobin/hydrophilic RTIL film	Hydrogen peroxide	Electrochemical sensor	Oxidative stress monitoring	160
Carbon-coated Ni magnetic NP-chitosan-DMF + RTIL	Heme proteins	Electrochemical sensor	Clinical & food analysis	161
Thionin-functionalized layered MoS_2_	DNA	Voltammetric sensor	Ultra-trace DNA detection	162
Superoxide dismutase/AuNP-chitosan-IL biocomposite	Superoxide anion	Amperometric biosensor	Oxidative stress monitoring	163

#### 3.5.4 Biomedical device enhancement

ILs are being explored for their potential to enhance the performance of medical devices. By incorporating ILs into hybrid materials, researchers can improve the functionality of biomedical tools used in diagnostics and treatment. For instance, ILs can stabilize nanoparticles in aqueous solutions, enhancing the dispersion of components like silk fibroin in hybrid devices.^164^

In medical device applications, ILs offer several advantages, including improved stability, biocompatibility, and mechanical properties. The use of ILs in hybrid materials could lead to the development of advanced medical devices with enhanced capabilities, particularly in clinical settings that require precise and efficient performance.^165^

Piperidinium-based ILs are emerging as key components in electrochemical devices. Their high thermal stability and ionic conductivity make them ideal for use as electrolytes in batteries, particularly lithium-ion batteries. By optimizing their cationic structure, researchers are working to enhance their ionic conductivity, improving battery performance and longevity. These advances are critical for developing energy storage solutions that minimize waste and reduce reliance on traditional energy sources.^166^

#### 3.5.5 Pharmaceutical applications

Ionic liquids (ILs) have gained growing importance in pharmaceutical and biomedical applications because of their exceptional chemical and thermal stability, structural tunability, and biocompatibility, enabling their use in drug synthesis, drug analysis, drug solubilization, and drug crystal engineering.^167^ Improving the solubility and bioavailability of poorly water-soluble drugs is a significant challenge in the pharmaceutical industry. Choline-based ILs, such as choline acetate, have shown considerable promise in enhancing the solubility of these drugs. These ILs work as solubilizing agents, improving drug absorption while preventing toxicity. In addition, choline-based ILs act as stabilizers for active pharmaceutical ingredients (APIs), preventing degradation during storage and transport and ensuring the efficacy of drugs.^168^

By converting conventional drugs into ionic-liquid forms (API-ILs), ILs provide controlled ion formation and tunable solvation in biological environments, offering a versatile strategy to overcome limitations in drug solubility, bioavailability, and polymorphism while advancing ionic-liquid-based pharmaceutics for broader chemical, biological, and medical applications.^169^

ILs are also increasingly used to enhance the performance of poorly soluble drugs by improving their solubility and stability in drug formulations. Furthermore, task-specific ILs (TSILs) provide a unique opportunity to create drug delivery systems that offer controlled release and targeted delivery, further advancing pharmaceutical applications.^170^

#### 3.5.6 Ionic liquids in organic synthesis and drug development

ILs have proven valuable in organic synthesis, particularly in the development of pharmaceutical drugs. Pyridinium-based ILs, for example, are used as catalysts in the synthesis of drug derivatives, such as 1,4-dihydropyridines and dihydropyrimidinones. Their ability to facilitate organic reactions, such as Friedel–Crafts and Grignard reactions, has significantly enhanced the efficiency of drug synthesis.^137^ Additionally, ILs promote more sustainable practices by reducing the need for hazardous solvents and reagents, making them an important tool in drug development.

The use of ILs in organic synthesis not only improves the efficiency of drug manufacturing but also contributes to the development of novel pharmaceutical compounds with better therapeutic outcomes. Ionic liquids are emerging as versatile and powerful tools in various biomedical applications, including drug delivery, tissue engineering, biosensors, and medical device enhancement. Their unique properties, such as high ionic conductivity, biocompatibility, and the ability to dissolve and modify natural polymers, make them highly promising for the development of innovative medical technologies.^171^ As research continues to explore and optimize their biomedical applications, ILs hold the potential to revolutionize the treatment of diseases, improve diagnostic tools, and enhance pharmaceutical formulations, ultimately leading to more effective, targeted, and safer therapies.

#### 3.5.7 Bio-ionic liquids (B-ILs)

Traditional ionic liquids (ILs), particularly those based on long alkyl chains like alkyl imidazolium and alkyl benzimidazolium, have raised environmental concerns due to their poor biodegradability and potential toxicity. These challenges have prompted the development of biodegradable ionic liquids (B-ILs), which are synthesized from renewable, bio-based precursors. B-ILs are designed to be environmentally benign, biodegradable, and exhibit low toxicity, making them suitable alternatives for applications in green chemistry, pharmaceuticals, and other industries focused on sustainability.^172^

B-ILs are primarily synthesized using bio-derived precursors, such as choline (ammonium) hydroxide cations and amino acid-based counter anions, following green chemistry principles. This approach minimizes the use of non-renewable resources and reduces the need for extensive chemical modifications, which are common in traditional IL synthesis. One well-known example is choline-based B-ILs, such as (2-hydroxyethyl)-ammonium lactate, which have been shown to possess high biodegradability (around 95% according to European standards) and low toxicity.^173^

The shift towards biodegradable ionic liquids (B-ILs) not only addresses the environmental and health concerns associated with conventional ILs but also promotes the adoption of sustainable technologies across diverse fields. For instance, B-ILs are increasingly applied in the chemical and pharmaceutical industries, where their biodegradable nature helps minimize ecological impact, while their low toxicity enhances safety in drug development, delivery systems, and other biomedical applications.^174^ However, the review emphasizes that although conventional ILs are considered “green solvents” due to their negligible vapor pressure, their high stability and persistence in aquatic and terrestrial environments pose significant eco-toxicological and human health risks, primarily through mechanisms such as cell membrane disruption and the induction of oxidative stress.^175^

**Fig. 9:**
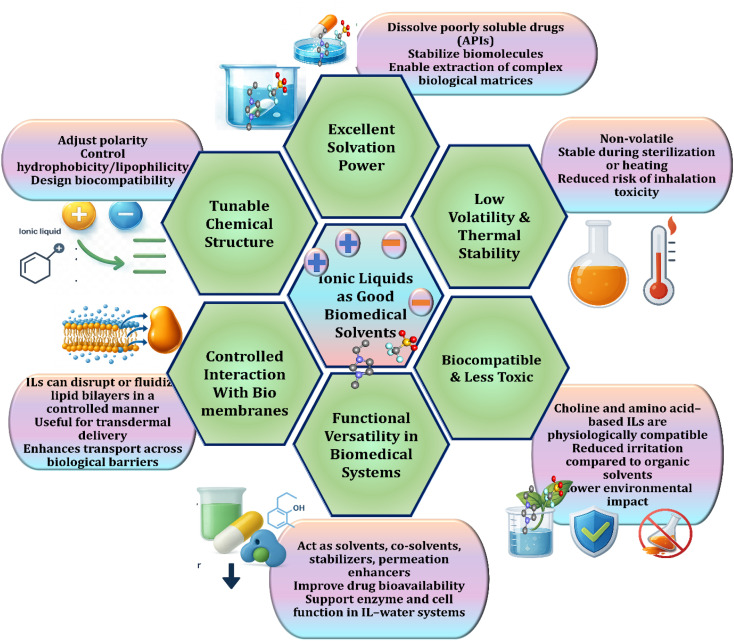
Overview of the key properties that make ionic liquids (ILs) effective biomedical solvents. Partially reproduced and modified from ref. 177, licensed under CC BY 4.0.

As the demand for eco-friendly alternatives in industrial and biomedical applications grows, B-ILs hold considerable promise for facilitating more sustainable production processes and advancing green chemistry. Their development aligns with global goals of reducing chemical waste, enhancing sustainability, and minimizing the ecological footprint of industrial practices, while maintaining the advantages of traditional ILs, such as their ionic conductivity and solubility in a wide range of substances. ILs can be structurally tuned *via* cation–anion combinations to modulate polarity, hydrophobicity, and biocompatibility. They offer strong solvation power for poorly soluble drugs and biomolecules, exhibit low volatility and high thermal stability, and demonstrate reduced toxicity and environmental impact compared to conventional organic solvents.^176^ Their multifunctionality enables roles as solvents, co-solvents, stabilizers, and permeation enhancers, and they interact controllably with biomembranes to facilitate drug transport across biological barriers. Fig. 9 shows schematic representation of the key properties that make ionic liquids effective biomedical solvents, including tunable chemical structure, excellent solvation power, low volatility and thermal stability, biocompatibility, functional versatility, and controlled interaction with biomembranes.

### 3.6 Agricultural applications: sustainable fertilizers and pesticides

Choline-based ionic liquids (ILs) are gaining attention in the agricultural sector for their potential to promote sustainable practices. A key area of interest is their use in the development of controlled-release fertilizers. Choline chloride-based ILs are used in the formulation of fertilizers that release nutrients gradually over time. This controlled release ensures that plants receive a consistent supply of essential nutrients, optimizing nutrient uptake and reducing the need for frequent fertilization. As a result, nutrient runoff, which is a major environmental issue associated with traditional fertilizers, is minimized. This not only improves nutrient efficiency but also helps to reduce the pollution of water systems caused by excessive fertilization.^178^

Choline-based and other novel ionic liquids (ILs) have diverse applications due to their tunable properties, biodegradability, and environmental compatibility. Table 5 summarizes the main types of novel ILs, representative examples, their unique features, and their key applications in agriculture, green chemistry, energy storage, and environmental remediation.

**Table 5:** Novel ionic liquids, their examples, key properties, and applications

Type of novel IL	Examples	Advantages	Applications	Ref.
Choline-based ILs	Choline chloride ([Ch][Cl]), choline acetate ([Ch][Ac])	Biodegradable, non-toxic, water-soluble, environmentally friendly	Controlled-release fertilizers, biopesticides, biomass processing, drug delivery	178
Ammonium-based ILs	Tetraalkylammonium salts, *e.g.*, [N_4_,_4_,_4_,_4_][Tf_2_N]	Thermally stable, biodegradable, non-volatile	Green catalysis, biodiesel production, energy storage, lubricants	179, and 180
Piperidinium-based ILs	1-Butyl-1-methylpiperidinium bis(trifluoromethylsulfonyl)imide ([BMPip][Tf_2_N])	High ionic conductivity, thermal stability, electrochemically stable	Electrolytes in lithium-ion batteries, supercapacitors, electrochemical devices	166
Imidazolium-based ILs	1-Butyl-3-methylimidazolium chloride ([Bmim][Cl]), 1-ethyl-3-methylimidazolium acetate ([Emim][Ac])	Tunable polarity, excellent solubility, antimicrobial activity	Catalysis, green solvents, CO_2_ capture, antimicrobial applications	106
Phosphonium-based ILs	Tetrabutylphosphonium bromide ([P_4_,_4_,_4_,_4_][Br])	High thermal & chemical stability, low viscosity	Lubricants, high-temperature catalysis, electrochemical devices	181, and 182
Task-specific ILs (TSILs)	Amino-functionalized ILs, hydroxyl-functionalized ILs	Designed for selective reactions, CO_2_ capture, enhanced solubility	CO_2_ sequestration, selective extraction, catalysis	183
Sugar- or amino acid-based ILs	Imidazolium amino acid ILs ([Emim][Gly]), choline-based sugar ILs	Biodegradable, low toxicity, biocompatible	Environmental remediation, antimicrobial agents, green chemistry	184

#### 3.6.1 Enhancing biopesticide efficiency

In addition to fertilizers, choline-based ILs are also being explored for their role in improving biopesticide efficacy. Biopesticides, which are derived from natural sources, provide a safer and more environmentally friendly alternative to traditional chemical pesticides. However, their effectiveness can sometimes be limited by issues related to solubility and stability. Choline-based ILs can enhance the solubility and stability of biopesticides, making them more effective in controlling pests while ensuring that they remain environmentally benign.^185^ This improvement in the performance of biopesticides contributes to more sustainable pest management solutions, reducing the need for harmful chemical pesticides and minimizing their impact on ecosystems.

By leveraging the properties of choline-based ILs, the agricultural industry can adopt greener, more efficient alternatives for both fertilization and pest control. These innovations support the shift toward sustainable agriculture, with benefits for both crop yield and environmental health.

### 3.7 Environmental applications: green remediation solutions

Biodegradable ionic liquids (ILs) offer a promising avenue for addressing environmental contamination. Their unique properties, such as selective interactions with pollutants, enable them to facilitate more efficient biodegradation. Traditional chemical agents and solvents often pose ecological risks, contributing to long-term environmental damage. In contrast, biodegradable ILs can be employed in the remediation of pollutants from soil, water, and air without the persistence and bioaccumulation issues associated with conventional chemicals.

Their potential as green remediation agents is rooted in their ability to function as environmentally friendly solvents. For example, imidazolium-based ILs, coupled with amino acid or sugar-based anions, exhibit antimicrobial properties and biocompatibility, making them suitable for agricultural and environmental applications. These ILs target microbial membranes selectively, minimizing human cell toxicity while accelerating biodegradation processes, a critical feature for environmental sustainability.^106^ This switch to biodegradable ILs in industrial and environmental cleanup efforts aligns with the global drive for sustainable practices and reducing the ecological footprint of industrial processes.

Green chemistry is a vital field that seeks to minimize the environmental impacts of chemical processes by developing more sustainable and energy-efficient alternatives. Ionic liquids (ILs) have gained widespread attention in this domain due to their non-volatile nature, recyclability, and ability to replace traditional solvents. These properties make ILs especially valuable in areas such as catalytic reactions, solvent systems, biomass processing, and CO_2_ capture.^186^

Traditional solvents, especially volatile organic compounds (VOCs), contribute significantly to air pollution and pose health risks. In contrast, ILs offer a safer, non-volatile alternative with low vapor pressures. Their recyclability further enhances their appeal as green solvents. Various reactions, including Friedel–Crafts and Grignard reactions, have demonstrated that ILs can enhance reaction efficiency while minimizing harmful solvent use.^187^ Additionally, ILs are useful in extracting bioactive compounds and essential oils, supporting sustainable practices in biotechnology and pharmaceuticals.

#### 3.7.1 Biomass processing and biofuel production

ILs have emerged as a crucial tool in biomass processing, particularly in biofuel production. Their ability to dissolve lignocellulosic biomass efficiently enables the extraction of fermentable sugars, which can be converted into bioethanol and biodiesel. This process provides a renewable alternative to fossil fuels, promoting sustainability. Studies have shown that ILs can break down complex biomass structures, offering a more energy-efficient and environmentally friendly route to biofuel production.^188^

The dissolution of lignocellulosic biomass in ILs involves intricate interactions between the solvent and the biomass components. ILs disrupt the hydrogen-bonding network within cellulose, leading to the solubilization of cellulose and partial depolymerization of lignin. This process enhances the accessibility of polysaccharides, such as cellulose and hemicellulose, to enzymatic hydrolysis, thereby improving the yield of fermentable sugars. The choice of IL, particularly the anion component, plays a crucial role in determining the efficiency of biomass dissolution and subsequent sugar release.^189^

The application of ionic liquids (ILs) in biomass processing offers several notable advantages. ILs effectively reduce the crystallinity of cellulose, enhancing its accessibility to enzymatic hydrolysis and thereby improving sugar yields. Certain ILs can also selectively solubilize lignin, enabling the separation and valorization of lignin as a valuable by-product. Additionally, ILs can be recovered and reused across multiple processing cycles, which reduces both the overall cost and the environmental impact of the process. Their non-volatile nature and thermal stability further contribute to environmentally compatible and sustainable biomass processing. Despite these benefits, several challenges remain, including the relatively high cost of ILs, potential toxicity to microorganisms used in downstream fermentation, and the need for efficient recovery and recycling strategies. Overcoming these limitations is crucial to ensuring the commercial viability of IL-based biomass processing technologies.^190^

For esterification, acidic ILs such as 1-butyl-3-methylimidazolium hydrogen sulfate ([BMIm][HSO_4_]) and sulfonic acid-functionalized imidazolium ILs provide strong Brønsted acidity, effectively converting high-FFA oils and waste cooking oils into biodiesel.^191,192^ In transesterification, basic ILs such as choline hydroxide ([Ch][OH], choline acetate [Ch][OAc]), and quaternary ammonium salts tetrabutylammonium hydroxide ([Bu_4_N][OH]) act as green, biodegradable catalysts that generate methoxide ions *in situ*, thereby promoting efficient triglyceride conversion.^193,194^

Additionally, imidazolium- and phosphonium-based ILs 1-butyl-3-methylimidazolium tetrafluoroborate ([BMIm][BF_4_]), 1-butyl-3-methylimidazolium hexafluorophosphate ([BMIm][PF_6_]), trihexyltetradecylphosphonium trifluoromethanesulfonate ([THTDPh][CF_3_SO_3_]) are valuable as co-solvents, enhancing oil–methanol miscibility and accelerating reaction kinetics.^195,196^ Guanidinium-based ILs, with their strong basicity, further broaden the scope by enabling biodiesel sy nthesis under low-temperature conditions.^197^

Overall, ILs offer multiple catalytic functions—acidic, basic, or amphiphilic—while also addressing limitations of conventional catalysts such as soap formation, poor reusability, and environmental hazards. Their versatility positions them as promising candidates for next-generation biodiesel processes. As summarized in Table 6, various ionic liquids have been explored as catalysts and co-solvents in biodiesel production, demonstrating roles ranging from Brønsted acidic esterification catalysts to basic transesterification promoters.

**Table 6:** Summarizes representative ionic liquids (ILs) applied in biodiesel production, highlighting their reaction type, catalytic role

Ionic liquid	Reaction type	Role in biodiesel production	References
[BMIm][HSO_4_] (1-butyl-3-methylimidazolium hydrogen sulfate)	Esterification	Acidic IL, catalyzes esterification of free fatty acids (FFAs) in high-FFA oils	191
[HSO_3_–Bmim][HSO_4_] (sulfonic acid functionalized imidazolium IL)	Esterification	Task-specific IL with strong Brønsted acidity, effective for waste cooking oil	192
[EMIm][OAc] (1-ethyl-3-methylimidazolium acetate)	Transesterification & Co-solvent	Enhances miscibility of oil–methanol, promotes FFA esterification	198
[BMIm][BF_4_] (1-butyl-3-methylimidazolium tetrafluoroborate)	Co-solvent	Improves methanol–oil mixing, accelerates reaction rate	199
[BMIm][PF_6_] (1-butyl-3-methylimidazolium hexafluorophosphate)	Co-solvent	Hydrophobic IL, enhances solubility of triglycerides	197
[Ch][OH] (choline hydroxide)	Transesterification	Biodegradable base catalyst, generates methoxide for triglyceride conversion	193
[Ch][OAc] (choline acetate)	Transesterification	Mild base catalyst, eco-friendly, effective for refined oils	194
[Bu_4_N][OH] (tetrabutylammonium hydroxide)	Transesterification	Quaternary ammonium IL, acts as strong base catalyst	195
Phosphonium ILs (*e.g.*, trihexyl(tetradecyl)phosphonium triflate [THTDPh][CF_3_SO_3_])	Transesterification	High thermal stability, effective in methanolysis of oils	199
Guanidinium-based ILs	Transesterification	Strongly basic, effective for low-temperature biodiesel synthesis	200

Hydrogen is a promising clean energy carrier, and lignocellulosic biomass offers a renewable feedstock for its production.^201^ While thermochemical routes such as gasification and pyrolysis provide high yields, biological processes like dark and photofermentation are milder but limited by slower rates and sugar accessibility. Ionic liquid (IL) pretreatment has emerged as an effective strategy to enhance microbial hydrogen production.^202^ For instance, 1-butyl-3-methylimidazolium chloride (BMIMCl)-regenerated cellulose increased hydrogen yields by tenfold compared to raw cellulose, while BMIMHSO_4_-pretreated *Arundo donax* L. biomass improved yields by approximately 35%. Differences in microbial tolerance to various ILs, such as 1-butyl-3-methylimidazolium acetate (BMIMAc) *versus* BMIMCl, highlight the role of ion–protein interactions.^203^ Overall, IL-assisted pretreatment significantly improves sugar availability and hydrogen production, offering a promising approach for sustainable bioenergy generation.

Choline-based ILs, recognized for their non-toxicity and biodegradability, are becoming increasingly important in green chemistry. These ILs are capable of solvate and stabilize a broad range of compounds, making them ideal for chemical processes such as enzyme catalysis, bioactive compound extraction, and controlled-release fertilizers. Their application in drug manufacturing and biodiesel production helps reduce the environmental impact of these industries, making them more sustainable.^179^

#### 3.7.2 Ammonium-based ionic liquids: key characteristics and applications

Ammonium-based ILs contribute significantly to green chemistry due to their biodegradability, non-toxicity, and potential for renewable sourcing. These ILs are used as catalysts or co-catalysts in organic synthesis, biocatalysis, and biodiesel production, offering enhanced reaction efficiency while minimizing waste and energy use. Their thermal and chemical stability allows for reactions to occur under milder conditions, further reducing the energy requirements for industrial processes. Furthermore, ammonium-based ILs are being explored for use in energy storage technologies, such as supercapacitors, batteries, and fuel cells, improving the performance and lifespan of electrochemical devices.^179^

#### 3.7.3 CO_2_ capture and sequestration with task-specific ionic liquids

One of the most promising applications of ionic liquids (ILs) in green chemistry is their role in carbon dioxide (CO_2_) capture and sequestration. Task-specific ionic liquids (TSILs), functionalized with groups such as amines or hydroxyls, can selectively bind CO_2_ through chemical interactions, including carbamate formation with amines or hydrogen bonding with hydroxyl groups. This mechanism enables high CO_2_ solubility and selectivity under mild conditions. By incorporating ILs into capture systems, CO_2_ can be efficiently extracted from flue gases or industrial emissions, providing a sustainable alternative to conventional solvents. Additionally, ILs are non-volatile and thermally stable, which minimizes environmental risks and allows for their reuse in multiple capture cycles. The tunability of ILs offers the possibility to optimize CO_2_ absorption capacity, kinetics, and recyclability, making them an effective tool for mitigating greenhouse gas emissions and advancing carbon management strategies.^183^

**Fig. 10:**
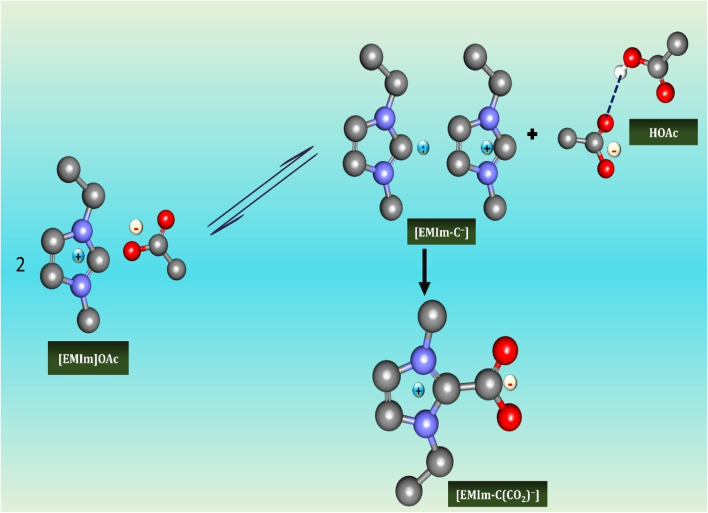
Proposed interaction pathways of CO_2_ with 1-ethyl-3-methylimidazolium acetate ([EMIm]OAc). Partially reproduced and modified from ref. 204, licensed under Creative Commons Attribution 4.0 International (CC BY 4.0).

The reaction of carbon dioxide (CO_2_) with 1-ethyl-3-methylimidazolium acetate ([EMIm]OAc) involves a combination of chemical and physical interactions that enable efficient CO_2_ capture (Fig. 10). When CO_2_ is introduced into the ionic liquid, it can form a complex with the C_2_–H of the imidazole ring, resulting in the EMIm–CO_2_ adduct. In this complex, CO_2_ becomes partially negatively charged, and its linear structure is disrupted, lowering the activation energy of the reaction and facilitating electron-mediated reduction. Simultaneously, the acetate anion (OAc^−^) acts as a nucleophile, abstracting the acidic proton from the C_2_ position of the imidazolium cation to generate an imidazolium carbene-like species, which then attacks the electrophilic carbon of CO_2_ to form a stable imidazolium carboxylate adduct.^204^ Alternatively, CO_2_ can directly react with the acetate anion to form an O-bound carbamate-like species. The imidazolium cation stabilizes these intermediates through hydrogen bonding, while the process remains reversible, allowing the ionic liquid to release CO_2_ upon heating or reduced CO_2_ pressure. Overall, [EMIm]OAc captures CO_2_ efficiently through both chemical bonding and physical solvation, making it a versatile medium for CO_2_ sequestration.^205^

#### 3.7.4 Green catalysis and sustainable chemical processes

ILs are integral to the development of green catalysis, where they serve as catalysts or co-catalysts in a wide range of reactions. Their tunable properties allow for the design of ILs that enhance reaction rates, selectivity, and yield while reducing energy consumption and waste generation. In biodiesel production, for example, ammonium-based ILs replace traditional catalysts, providing a cleaner, more sustainable alternative. These advances contribute to the transition to a sustainable chemical industry by improving the efficiency and environmental impact of industrial reactions^206^

#### 3.7.5 Lubricant applications: green alternatives for energy efficiency

Ammonium-based ILs are also used as lubricants in mechanical systems. These ILs reduce friction and wear, even under extreme pressure and temperature conditions, making them ideal for energy-efficient lubrication systems. For example, quaternary ammonium ILs are used in gear systems, where they outperform conventional lubricants by reducing energy losses and improving system efficiency.^180^ These lubricants are not only more efficient but are also non-toxic, non-volatile, and biodegradable, providing a green alternative to traditional lubricants.

#### 3.7.6 The future of ionic liquids in green chemistry

Ionic liquids are playing an increasingly important role in advancing green chemistry by providing sustainable, environmentally friendly alternatives to traditional solvents, catalysts, and materials. Their applications in biomass processing, CO_2_ capture, and green catalysis are helping to reduce waste and energy consumption while minimizing the use of toxic chemicals. As research continues to explore new formulations and applications for ILs, their potential to support sustainable chemical processes and contribute to environmental protection will only increase. The development of more efficient and eco-friendly chemical processes positions ILs at the forefront of shaping the future of green chemistry.^206^

Biodegradable ILs represent a breakthrough in sustainable chemistry, offering innovative solutions for environmental remediation, green catalysis, and sustainable energy storage. Their use in biomass processing, CO_2_ capture, and as eco-friendly solvents and lubricants highlights their versatility and potential for addressing a wide range of environmental and industrial challenges. As research in this field progresses, biodegradable ILs are poised to play a significant role in shaping a more sustainable and environmentally responsible future.^207^

### 3.8 Solvent systems for chemical reactions

#### 3.8.1 The role of piperidinium-based ionic liquids

Piperidinium-based ionic liquids (ILs) have gained significant attention in recent years due to their unique properties, particularly their ability to act as solvents in a wide range of chemical reactions. These ILs are especially effective in processes involving polar compounds, making them versatile and ideal for synthetic reactions that require solvation of both polar and non-polar substances. Their non-volatile nature and high solvation capacity offer substantial advantages over traditional organic solvents, which often exhibit volatility, toxicity, or environmental concerns.^208^

One of the key benefits of piperidinium-based ILs is their ability to replace conventional organic solvents in chemical reactions where toxicity and volatility are issues. Traditional solvents can be harmful to both the environment and human health, and their disposal can lead to significant contamination. In contrast, piperidinium-based ILs offer a safer, non-volatile, and more sustainable alternative. This has positioned them as a valuable tool in the advancement of greener chemistry practices, aligning with the chemical industry's increasing focus on sustainability, safety, and reduced environmental impact.^209^

These ILs have been applied in various synthetic processes, especially in reactions where traditional solvents may not be suitable. Their use in chemical reactions has not only improved the efficiency of these processes but has also helped address the growing demand for greener, more eco-friendly practices in industrial chemistry.

#### 3.8.2 Metathesis reactions and synthesis of cationic ionic liquids (C-ILs)

An exciting application of ionic liquids, particularly piperidinium- and tetraethylammonium-based ILs, is in metathesis reactions. One example is the metathesis reaction involving tetraethylammonium hydroxide and various amino acids, which leads to the formation of C-ILs (cationic ionic liquids). These ILs are synthesized from tetraethylammonium-based cations and amino acid-derived anions, such as asparaginate, glutamate, and isoleucinate.^210^

**Fig. 11:**
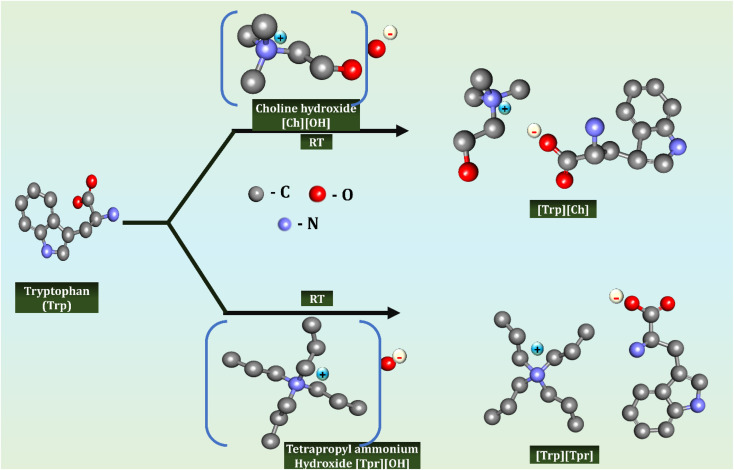
Schematic representation of the reaction mechanism for amino acid-based ionic liquid (IL) formation. Partially reproduced and modified from ref. 213, licensed under CC BY 4.0.

The amino acid-based ILs were synthesized following literature procedures.^211,212^ The synthesis involved the neutralization of [Try] with equimolar quaternary ammonium compounds ([Ch] and [Tpr]) at ambient temperature and atmospheric pressure, as illustrated in Fig. 11. The synthesis of amino acid-based ionic liquids (ILs) occurs through a simple acid-base neutralization mechanism between tryptophan and quaternary ammonium hydroxides ([Ch][OH] or [Tpr][OH]). In the first step, the hydroxide ion (OH^−^) abstracts a proton from the carboxylic acid group (–COOH) of tryptophan, leading to the formation of a carboxylate anion (–COO^−^) with water as the only byproduct. Subsequently, the resulting tryptophan carboxylate anion associates electrostatically with the positively charged quaternary ammonium cation, yielding the desired amino acid-based ILs, [Try][Ch] and [Try][Tpr].^213^ This mechanism underscores the efficiency and sustainability of the process, as it proceeds under mild conditions without the need for catalysts, metal salts, or halide exchange steps, and produces only water as a benign byproduct. The strong ionic interactions between the tryptophan anion and quaternary ammonium cations ensure the stability of the ILs, while the modular nature of this method enables the design of diverse ILs by varying the amino acid or cation.^214^ Overall, this clean and versatile synthetic route aligns well with the principles of green chemistry and offers a promising alternative to conventional IL synthesis methods.^215^

This approach is particularly attractive for its environmental and economic benefits. Unlike traditional purification methods that involve metal salts, halide pollutants, or ion-exchange steps, the metathesis reaction for C-ILs does not require these harmful contaminants. The reaction produces pure byproducts, including water (H_2_O), making it a cleaner, more sustainable method for IL synthesis.^216^ This process not only eliminates the need for potentially hazardous substances but also reduces the environmental impact of chemical synthesis, making it a cost-effective and eco-friendly alternative to conventional methods.

The growing use of piperidinium-based and tetraethylammonium-based ILs in chemical reactions highlights their potential to revolutionize the chemical industry. Their non-toxic, non-volatile nature, alongside their ability to enhance reaction efficiency and sustainability, positions them as essential components in the development of greener, more sustainable solvent systems.^217^ The ability to synthesize C-ILs through metathesis reactions further demonstrates their versatility and the promise they hold for advancing eco-friendly chemistry practices.^218^ Owing to their exceptional tunability, stability, and negligible environmental footprint, ionic liquids (ILs) are emerging as transformative designer materials capable of driving sustainable innovation across material synthesis, energy systems, gas capture, and biomass processing, representing a paradigm shift in green chemistry and industrial technology^219^ when supported by rigorous research and academia–industry collaboration.

## 4. Conclusion

The development of biocompatible ionic liquids represents a significant step forward in the search for materials that can simultaneously meet the demands of green chemistry and modern medicine. Once regarded primarily as safer alternatives to hazardous solvents, ILs are now recognized as multifunctional agents with the ability to act as catalysts, stabilizers, and therapeutic platforms. Their adaptability is exemplified by the creation of ionic liquid-based nanoparticles, which integrate the chemical versatility of ILs with the unique advantages of nanomaterials. These hybrid systems hold great promise in drug delivery, antimicrobial therapies, anticancer treatments, tissue engineering, and biosensing, where stability, selectivity, and controlled release are critical. Furthermore, the concept of “designer ILs,” through task-specific and bio-ionic formulations, enables researchers to fine-tune their properties to optimize biocompatibility and performance. However, challenges remain—cytotoxicity must be carefully managed, biodegradability must be improved, and cost-effective large-scale production must be realized before ILs can fully transition from laboratory research to widespread industrial and clinical use. Looking ahead, the convergence of IL chemistry with nanotechnology, materials science, and biomedical engineering presents exciting opportunities to create sustainable solutions that address pressing global challenges. Biocompatible ILs and ILNs, with their dual capacity to advance both environmental sustainability and therapeutic innovation, may become key enablers of a future where green chemistry and human health progress hand in hand.

## Conflicts of interest

The authors declare that there is no conflict of interest.

## Data Availability

We confirm that this manuscript is a review article. No primary research results, datasets, software, or code have been generated, analysed, or reported as part of this work. Therefore, there are no associated research data to make available.
